# Intra-Amniotic Infection with *Ureaplasma parvum* Causes Preterm Birth and Neonatal Mortality That Are Prevented by Treatment with Clarithromycin

**DOI:** 10.1128/mBio.00797-20

**Published:** 2020-06-23

**Authors:** Kenichiro Motomura, Roberto Romero, Yi Xu, Kevin R. Theis, Jose Galaz, Andrew D. Winters, Rebecca Slutsky, Valeria Garcia-Flores, Chengrui Zou, Dustyn Levenson, Robert Para, Madison M. Ahmad, Derek Miller, Chaur-Dong Hsu, Nardhy Gomez-Lopez

**Affiliations:** a Perinatology Research Branch, Division of Obstetrics and Maternal-Fetal Medicine, Division of Intramural Research, *Eunice Kennedy Shriver* National Institute of Child Health and Human Development, National Institutes of Health, U.S. Department of Health and Human Services, Bethesda, Maryland, and Detroit, Michigan, USA; b Department of Obstetrics and Gynecology, Wayne State University School of Medicine, Detroit, Michigan, USA; c Department of Obstetrics and Gynecology, University of Michigan, Ann Arbor, Michigan, USA; d Department of Epidemiology and Biostatistics, Michigan State University, East Lansing, Michigan, USA; e Center for Molecular Medicine and Genetics, Wayne State University, Detroit, Michigan, USA; f Detroit Medical Center, Detroit, Michigan, USA; g Department of Obstetrics and Gynecology, Florida International University, Miami, Florida, USA; h Department of Biochemistry, Microbiology, and Immunology, Wayne State University School of Medicine, Detroit, Michigan, USA; University of Oklahoma Health Sciences Center

**Keywords:** *Ureaplasma*, antibiotics, bacterial burden, intra-amniotic infection, neonate, pregnancy, preterm labor

## Abstract

Intra-amniotic infection is strongly associated with adverse pregnancy and neonatal outcomes. Most intra-amniotic infections are due to *Ureaplasma* species; however, the pathogenic potency of these genital mycoplasmas to induce preterm birth is still controversial. Here, we first laid out a taxonomic characterization of *Ureaplasma* isolates from women with intra-amniotic infection, which revealed that Ureaplasma parvum is the most common bacterium found in this clinical condition. Next, using animal models, we provided a causal link between intra-amniotic inoculation with *Ureaplasma* species and preterm birth. Importantly, the intra-amniotic inoculation of *Ureaplasma* species induced high rates of mortality in both preterm and term neonates. The *in vivo* potency of U. parvum to induce preterm birth was not associated with known virulence factors. However, term-derived and preterm-derived U. parvum isolates were capable of inducing an intra-amniotic inflammatory response. Both U. parvum isolates invaded several fetal tissues, primarily the fetal lung, and caused fetal inflammatory response syndrome. This bacterium was also detected in the placenta, reproductive tissues, and most severely in the fetal membranes, inducing a local inflammatory response that was replicated in an *in vitro* model. Importantly, treatment with clarithromycin, a recently recommended yet not widely utilized antibiotic, prevented the adverse pregnancy and neonatal outcomes induced by U. parvum. These findings shed light on the maternal-fetal immunobiology of intra-amniotic infection.

## Abstract (precis)

Preterm birth is the leading cause of neonatal morbidity and mortality worldwide. Multiple etiologies are associated with preterm birth; however, 25% of preterm infants are born to a mother with intra-amniotic infection, most commonly due to invasion of the amniotic cavity by *Ureaplasma* species. Much research has focused on establishing a link between *Ureaplasma* species and adverse pregnancy/neonatal outcomes; however, little is known about the taxonomy of and host response against *Ureaplasma* species. Here, we applied a multifaceted approach, including human samples, *in vivo* models, and *in vitro* manipulations, to study the maternal-fetal immunobiology of *Ureaplasma* infection during pregnancy. Furthermore, we investigated the use of clarithromycin as a treatment for this infection. Our research provides translational knowledge that bolsters scientific understanding of *Ureaplasma* species as a cause of adverse pregnancy/neonatal outcomes and gives strong evidence for the use of clarithromycin as the recommended treatment for women intra-amniotically infected with *Ureaplasma* species.

## INTRODUCTION

Microbial-associated intra-amniotic inflammation, also known as intra-amniotic infection, is associated with a wide array of adverse pregnancy and neonatal outcomes such as clinical chorioamnionitis (1), funisitis (2), bacteremia (3), and importantly, preterm labor and birth (4)- a syndrome that is the leading cause of neonatal morbidity and mortality worldwide (5, 6). The most frequently identified bacteria in amniotic fluid of women with intra-amniotic infection are *Ureaplasma* species, followed by *Mycoplasma hominis*, Gardnerella vaginalis and Streptococcus agalactiae, among others (1, 7–11). *Ureaplasma* has been classified into two species containing 14 serovars: Ureaplasma parvum (comprised of serovars 1, 3, 6, and 14) and Ureaplasma urealyticum (comprised of serovars 2, 4, 5, and 7 to 13) (12, 13). These two species are distinguishable based on differing genomic characteristics such as 16S rRNA gene sequence identity as well as unique and specific genes such as the multiple banded antigen and urease (14). Both *U. parvum* and *U. urealyticum* have been identified in amniotic fluid of women with intra-amniotic infection (13). However, the immune responses triggered by *Ureaplasma* species in the mother and the offspring have been poorly characterized.

Clinical studies have shown that women intra-amniotically infected with *Ureaplasma* species exhibit a more intense inflammatory response than those infected with other microorganisms (15). Moreover, the earlier the gestational age, the greater the intensity of the intra-amniotic inflammatory response induced by *Ureaplasma* species (16). However, not all women intra-amniotically infected with *Ureaplasma* species deliver preterm (17). This finding is consistent with animal studies in which the intra-amniotic administration of *Ureaplasma* species does not always lead to preterm birth. Initial studies using an established model of chronically catheterized rhesus macaques (18) showed that intra-amniotic inoculation with *U. parvum* or Mycoplasma hominis induces preterm labor, intra-amniotic inflammation, inflammation of the placenta (e.g., acute chorioamnionitis), and a systemic fetal inflammatory response (19). However, the ultrasound-guided intra-amniotic injection of *U. parvum* in rhesus macaques does not induce preterm labor or a severe fetal inflammatory response but causes intra-amniotic and uterine inflammation (20). The difference between the two macaque models could be explained by the distinct methodologies utilized. In mice, surgical intra-amniotic infection with *Ureaplasma* species does not cause preterm birth (21). Thus, we hypothesized that there is diversity in the *Ureaplasma* species found in the amniotic cavity of women with intra-amniotic infection, which differentially modulate the host immune responses of the mother and the fetus and may dictate pregnancy outcomes.

In the present study, we isolated and cultivated *Ureaplasma* species from amniotic fluid of women with intra-amniotic infection and performed 16S rRNA gene and whole-genome sequencing to provide an in-depth characterization of these isolates. The *Ureaplasma* isolates were then utilized to create a murine model of *Ureaplasma*-induced intra-amniotic infection to evaluate pregnancy and neonatal outcomes, localize sites of bacterial invasion, and evaluate maternal and fetal immune responses. Finally, we evaluated whether clarithromycin, a clinically relevant antibiotic (22–24), is an effective treatment for intra-amniotic infection caused by *Ureaplasma* species.

## RESULTS

### Taxonomic analysis of *Ureaplasma* isolates from women with intra-amniotic infection.

**FIG 1:**
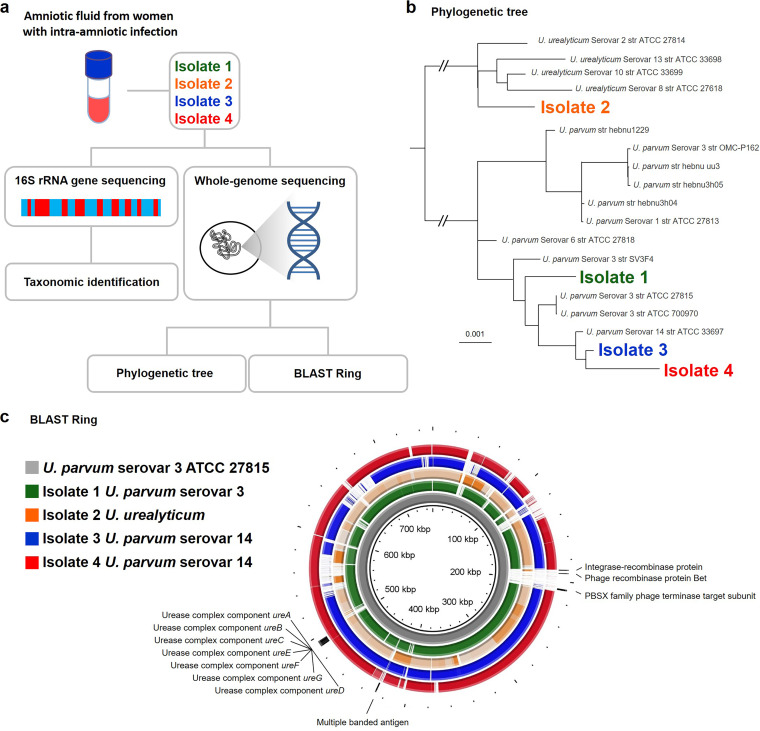
Taxonomic analysis of *Ureaplasma* isolates from women with intra-amniotic infection. (a) Experimental design for the 16S rRNA gene sequencing and whole-genome sequencing of bacteria isolated from amniotic fluid of women with intra-amniotic infection (*n* = 4). (b) Whole-genome single nucleotide polymorphism (SNP) phylogeny of four clinical isolates and 15 publicly available *Ureaplasma* genomes inferred using whole-genome maximum-likelihood phylogeny under a generalized time-reversible model. The tree was rooted at the midpoint. The scale bar indicates the number of substitutions per nucleotide position. (c) Representation (BLAST Ring) of the four isolate genomes using *U. parvum* serovar 3 ATCC 27815 (CP000942) as the reference. Known virulence genes of *Ureaplasma* species are shown.

First, we explored whether there were taxonomic differences in the *Ureaplasma* isolates from women with intra-amniotic infection. DNA was extracted from four *Ureaplasma* isolates from women with intra-amniotic infection (the clinical and demographic characteristics of these women are shown in Table S1 in the supplemental material) and 16S rRNA gene sequencing was performed (Fig. 1a). The 16S rRNA gene sequences of isolate 1, isolate 3, and isolate 4 were identical to those of *U. parvum* serovar 3 and serovar 14 (Table 1). Specifically, the trimmed 16S rRNA gene sequences (1,025 bp aligning with nucleotide positions 344101 to 345125 of the genome of *U. parvum* serovar 3 ATCC 27815, accession no. CP000942.1) for isolates 1, 3, and 4 were identical to *U. parvum* serovar 3 (ATCC 27815) or serovar 14 (ATCC 33697) (Table 1). The 16S rRNA gene sequence of isolate 2 (1,021 bp) closely matched that of *U. urealyticum* serovars 2, 8, or 10 (ATCC 27814, ATCC 27618, and ATCC 33699, respectively) (Table 1). Whole-genome sequencing was then performed to further characterize the taxonomic profile of each *Ureaplasma* isolate (Fig. 1a). The phylogenetic tree based on genomic single nucleotide polymorphism (SNP) data indicated a clear division of two main clusters, with one cluster containing all *U*. *parvum* serovars (1, 3, 6, and 14) and the other containing all *U*. *urealyticum* serovars (2, 8, 10, and 13) (Fig. 1b). Within the subclusters, isolate 1 grouped with multiple serovar 3 strains, and isolates 3 and 4 grouped with the serovar 14 strain (Fig. 1b). Isolate 2 was not positioned with any specific *U*. *urealyticum* serovar (Fig. 1b). Based on these clusters, we determined that the taxonomic identity of isolate 1 corresponded to *U*. *parvum* serovar 3, and the taxonomic identities of both isolates 3 and 4 corresponded to *U*. *parvum* serovar 14. The taxonomic identity of isolate 2 was determined to correspond with *U. urealyticum* of an unknown serovar. To further explore the differences between *Ureaplasma* species isolated from women with intra-amniotic infection, DNA sequence analysis of known virulence genes (urease complex and multiple banded antigen [25]) was performed. No substantial differences were observed between the four isolates (Fig. 1c). Specifically, the similarity of the urease gene complex of the three *U*. *parvum* isolates ranged from 99.95 to 99.96% (Fig. 1c). As expected, the similarity of the urease gene complex sequences of the *U*. *urealyticum* isolate was only between 90.86 and 90.88% to those of the *U*. *parvum* isolates (Fig. 1c). In addition, we evaluated the putative pathogenicity island, a 20-kb region variably present in serovars 1, 3, and 6 that contains potential integrase/recombinase genes with phage orthologs (26), in all isolates. However, this pathogenicity island was not present in any of the genomes of the four isolates (Fig. 1c). Together, these genomic analyses revealed that the taxonomic identity of *Ureaplasma* species isolated from women with intra-amniotic infection is diverse and yet not distinguishable by known virulence genes. Importantly, *U. parvum* serovar 14 was present in both a woman who delivered preterm and a woman who delivered at term, suggesting that taxonomy cannot solely account for adverse pregnancy outcomes caused by this genital mycoplasma.

**TABLE 1:** Percent similarity of 16S rRNA gene sequences of the isolates to publicly available *Ureaplasma* type strains

Isolate	% similarity to *U. parvum* serovars				% similarity to *U. urealyticum* serovars			
	Serovar 1, ATCC 27813 (ASM17137v1)	Serovar 3, ATCC 27815 (ASM1934v1)	Serovar 6, ATCC 27818 (ASM16989v1)	Serovar 14, ATCC 33697 (ASM17135v1)	Serovar 2, ATCC 27814 (ASM17155v1)	Serovar 8, ATCC 27618 (ASM16953v1)	Serovar 10, ATCC 33699 (ASM2126v1)	Serovar 13, ATCC 33698 (ASM17139v1)
1	99.90	100.00	99.90	100.00	98.83	98.83	98.83	98.73
2	98.73	98.83	98.73	98.83	99.90	99.90	99.90	99.80
3	99.90	100.00	99.90	100.00	98.83	98.83	98.83	98.73
4	99.90	100.00	99.90	100.00	98.83	98.83	98.83	98.73

10.1128/mBio.00797-20.9 TABLE S1 Clinical and demographic characteristics of women from whose amniotic fluid *Ureaplasma* species were isolated. Download Table S1, DOCX file, 0.01 MB. Copyright © 2020 Motomura et al. 2020 Motomura et al. This content is distributed under the terms of the Creative Commons Attribution 4.0 International license.

### *Ureaplasma* isolates from women with intra-amniotic infection can induce preterm birth and adverse neonatal outcomes.

**FIG 2:**
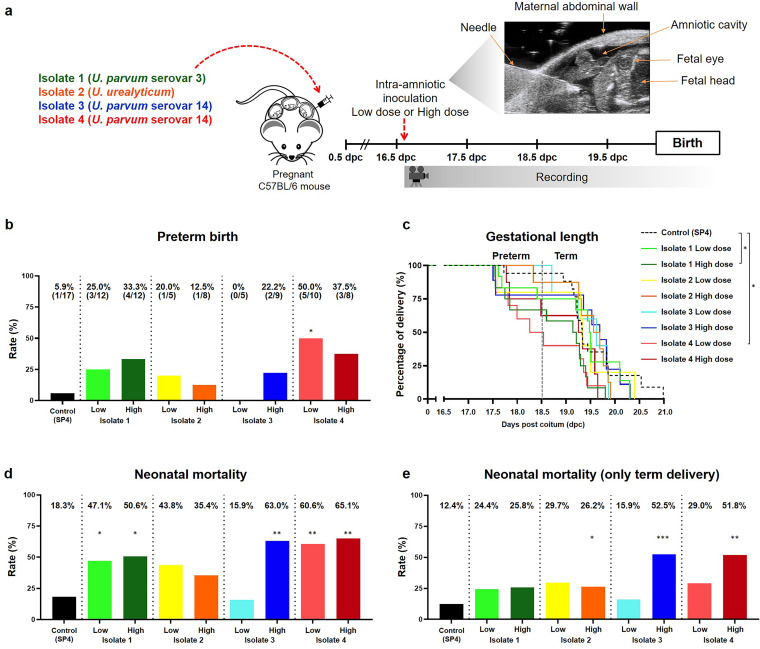
Preterm birth and adverse neonatal outcomes induced by intra-amniotic inoculation with *Ureaplasma* isolates. (a) Experimental design for the intra-amniotic inoculation with *Ureaplasma* species isolated from amniotic fluid of women with intra-amniotic infection (positive amniotic fluid culture and an IL-6 concentration of ≥2.6 ng/ml). Dams were intra-amniotically inoculated with *Ureaplasma* isolates (low dose [1 × 10^3^ to 1 × 10^4^ cells/25 μl] or high dose [1 × 10^4^ to 1 × 10^5^ cells/25 μl], *n* = 5 to 12 each) or injected with vehicle control (SP4 broth, 25 μl, *n* = 17) in each amniotic sac under ultrasound guidance at 16.5 days *post coitum* (dpc) and monitored until delivery. (b) Rates of preterm birth of dams intra-amniotically inoculated with *Ureaplasma* isolates or injected with control. The *P* values were determined by Fisher's exact tests. (c) Kaplan-Meier survival curves showing the gestational lengths at delivery of dams intra-amniotically inoculated with *Ureaplasma* isolates or injected with control. The *P* values were determined by Gehan-Breslow-Wilcoxon tests. (d) Mortality of neonates born to dams intra-amniotically inoculated with *Ureaplasma* isolates or injected with control. (e) Mortality of only those neonates born at term to dams intra-amniotically inoculated with *Ureaplasma* isolates or injected with control. The *P* values in panels (d) and (e) were determined by unpaired *t* tests. *, *P* < 0.05; **, *P* < 0.01; ***, *P* < 0.001.

Genomic analysis revealed that there are taxonomic differences in the *Ureaplasma* isolates obtained from women with intra-amniotic infection. Next, we evaluated whether these four isolates could induce different pregnancy outcomes in mice. Dams were intra-amniotically inoculated under ultrasound guidance in each amniotic sac with a low dose (from ≥1 × 10^3^ to ≤1 × 10^4^ cells/sac) or a high dose (from >1 × 10^4^ to ≤1 × 10^5^ cells/sac) of *Ureaplasma* species from one of the four clinical isolates and observed until delivery (Fig. 2a). These doses were chosen based on the concentrations of *Ureaplasma* species recovered from women with intra-amniotic infection. Controls were intra-amniotically injected with sterile 1× phosphate-buffered saline (PBS) or SP4 broth (vehicle control) (see Fig. S1 in the supplemental material). Each clinical *Ureaplasma* isolate induced a variable rate of preterm birth, with isolate 3 being the least effective at the low dose and isolate 4 showing the greatest potency at the low dose (Fig. 2b). Notably, intra-amniotic inoculation with isolate 4 (low dose) induced the highest rate of preterm birth compared to vehicle controls (Fig. 2b). Survival curve analysis indicated that isolate 1 (high dose) and isolate 4 (low dose) decreased the gestational length compared to vehicle controls (Fig. 2c). No differences in the rates of preterm birth or gestational length were observed between PBS- and vehicle control-injected dams (Fig. S1a and S1b).

10.1128/mBio.00797-20.1 FIG S1 Pregnancy outcomes of dams injected with PBS or SP4 broth. (a) Rates of preterm birth of dams intra-amniotically injected with PBS or SP4 broth. The *P* values were determined by a Fisher's exact test. (b) Kaplan-Meier survival curve showing the gestational ages at delivery of dams intra-amniotically injected with PBS or SP4 broth. Statistical analysis was performed using a Gehan-Breslow-Wilcoxon test. (c) Mortality rates of neonates born to dams intra-amniotically injected with PBS or SP4 broth. Statistical analysis was performed using an unpaired *t* test. (d) Mortality rates of only those neonates born at term to dams intra-amniotically injected with PBS or SP4 broth. Statistical analysis was performed using an unpaired *t* test. The results of all statistical analyses were non-significant. Download FIG S1, TIF file, 1.3 MB. Copyright © 2020 Motomura et al. 2020 Motomura et al. This content is distributed under the terms of the Creative Commons Attribution 4.0 International license.

Intra-amniotic infection is not only associated with preterm birth but also with adverse neonatal outcomes, even in term neonates (27). Therefore, we also evaluated the mortality rates of neonates born to dams intra-amniotically inoculated with clinical isolates of *Ureaplasma* species. A significantly elevated rate of mortality was observed for neonates born to dams inoculated with isolate 1 and isolate 4 (high and low doses) as well as the high dose of isolate 3 (Fig. 2d). When considering only neonates that were delivered at term, significantly higher rates of mortality were still observed for those neonates born to dams inoculated with a high dose of *Ureaplasma* isolates 2, 3, and 4 (Fig. 2e). The rates of neonatal mortality were comparable between PBS and vehicle controls (Fig. S1c and S1d).

Collectively, these results indicate that intra-amniotic inoculation with clinically isolated *Ureaplasma* species induces variable rates of preterm birth and neonatal mortality. Importantly, *U. parvum* serovar 14 isolated from a woman who delivered preterm (isolate 4) induced higher rates of preterm birth in mice than that from a woman who delivered at term (isolate 3), yet both isolates were associated with intra-amniotic inflammation (an interleukin-6 [IL-6] concentration of ≥2.6 ng/ml [28]) and acute maternal and fetal inflammatory responses in the placenta (Table S1). These data indicate that, although *U. parvum* serovar 14 displays variable *in vivo* potency to induce preterm birth, this bacterium consistently causes damage to the fetus and at the maternal-fetal interface (e.g., chorioamniotic membranes and decidua). Therefore, we next investigated the host immune responses triggered by both isolates of *U. parvum* serovar 14 in the amniotic cavity, in the fetus, and at the maternal-fetal interface.

### Intra-amniotic inoculation with *U. parvum* serovar 14 induces an intra-amniotic inflammatory response but not a maternal inflammatory response.

**FIG 3:**
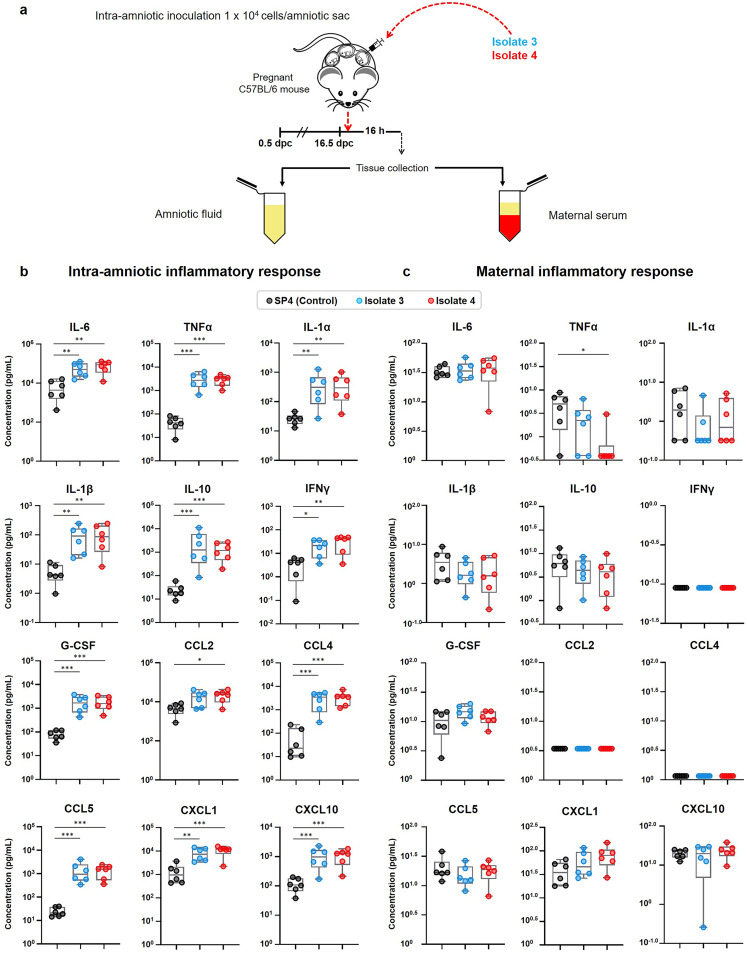
Intra-amniotic and maternal systemic inflammatory responses after intra-amniotic inoculation with U. parvum serovar 14. (a) Experimental design for the evaluation of intra-amniotic and maternal systemic inflammatory responses after intra-amniotic inoculation with two clinical isolates of *U. parvum* serovar 14. Dams were intra-amniotically inoculated with *U. parvum* isolate 3 (1.0 × 10^4^ cells/25 μl) or *U. parvum* isolate 4 (1.0 × 10^4^ cells/25 μl), or injected with vehicle control (SP4 broth, 25 μl) at 16.5 days *post coitum* (dpc) (*n* = 6 each). Amniotic fluid and maternal serum samples were collected 16 h after intra-amniotic inoculation for cytokine/chemokine multiplex analysis. (b and c) Cytokine/chemokine concentrations in amniotic fluid (b) and maternal serum (c). The data are shown as box-and-whisker plots, where midlines indicate medians, boxes indicate interquartile ranges, and whiskers indicate minimum and maximum ranges. The *P* values were determined by one-way analysis of variance (ANOVA), followed by Tukey’s tests to determine differences between groups. *, *P* < 0.05; **, *P* < 0.01; ***, *P* < 0.001.

It is well documented that women with *Ureaplasma*-associated intra-amniotic infection display a severe intra-amniotic inflammatory response (15). Therefore, we next evaluated whether intra-amniotic inoculation with low doses of isolates 3 and 4 (both *U. parvum* serovar 14) induced inflammatory responses in the amniotic cavity. Given that the intra-amniotic inflammatory response in women with *Ureaplasma*-associated intra-amniotic infection is subclinical in nature (29), we also determined the inflammatory response in the maternal circulation. Dams were intra-amniotically inoculated with either isolate 3 or isolate 4 of *U. parvum* serovar 14, and both amniotic fluid and maternal serum were collected (Fig. 3a). The amniotic fluid concentrations of IL-6, a cytokine used for the clinical diagnosis of intra-amniotic inflammation (28), were increased in dams inoculated with isolate 3 and isolate 4 compared to vehicle controls (Fig. 3b). Other cytokines upregulated in women with intra-amniotic infection (30) such as tumor necrosis factor alpha (TNFα), IL-1α, IL-1β, IL-10, gamma interferon (IFNγ), granulocyte colony-stimulating factor (G-CSF), CCL2, CCL4, CCL5, CXCL1, and CXCL10 were also increased in amniotic fluid from dams inoculated with isolate 3 or isolate 4 compared to vehicle controls (Fig. 3b). No differences were observed between isolate 3 and isolate 4 (Fig. 3b). Conversely, when we evaluated the maternal systemic inflammatory response, none of the cytokines were increased upon inoculation with isolate 3 or isolate 4 compared to vehicle controls (Fig. 3c). Cytokine concentrations in amniotic fluid and sera of dams injected with PBS and vehicle control are shown in Fig. S2 and S3.

10.1128/mBio.00797-20.2 FIG S2 Cytokine and chemokine concentrations in amniotic fluid from dams intra-amniotically injected with PBS or SP4 broth. The data are shown as box-and-whisker plots, where midlines indicate medians, boxes indicate interquartile ranges, and whiskers indicate minimum and maximum ranges. *P* values were determined by using unpaired *t* tests. *, *P* < 0.05; **, *P* < 0.01. Download FIG S2, TIF file, 0.1 MB. Copyright © 2020 Motomura et al. 2020 Motomura et al. This content is distributed under the terms of the Creative Commons Attribution 4.0 International license.

10.1128/mBio.00797-20.3 FIG S3 Cytokine and chemokine concentrations in maternal serum from dams intra-amniotically injected with PBS or SP4 broth. The data are shown as box-and-whisker plots, where midlines indicate medians, boxes indicate interquartile ranges, and whiskers indicate minimum and maximum ranges. Statistical analysis was performed by using unpaired *t* tests. The results of all statistical analyses were non-significant. Download FIG S3, TIF file, 0.1 MB. Copyright © 2020 Motomura et al. 2020 Motomura et al. This content is distributed under the terms of the Creative Commons Attribution 4.0 International license.

Together, these results show that intra-amniotic inoculation with either isolate of *U. parvum* serovar 14 induces a similar intra-amniotic inflammatory response in the absence of a maternal systemic inflammatory response. This finding indicated that a deeper characterization of the immunological effects of *U. parvum* serovar 14 on the fetus was required.

### *Ureaplasma parvum* serovar 14 invades the fetal organs, inducing fetal inflammatory response syndrome.

**FIG 4:**
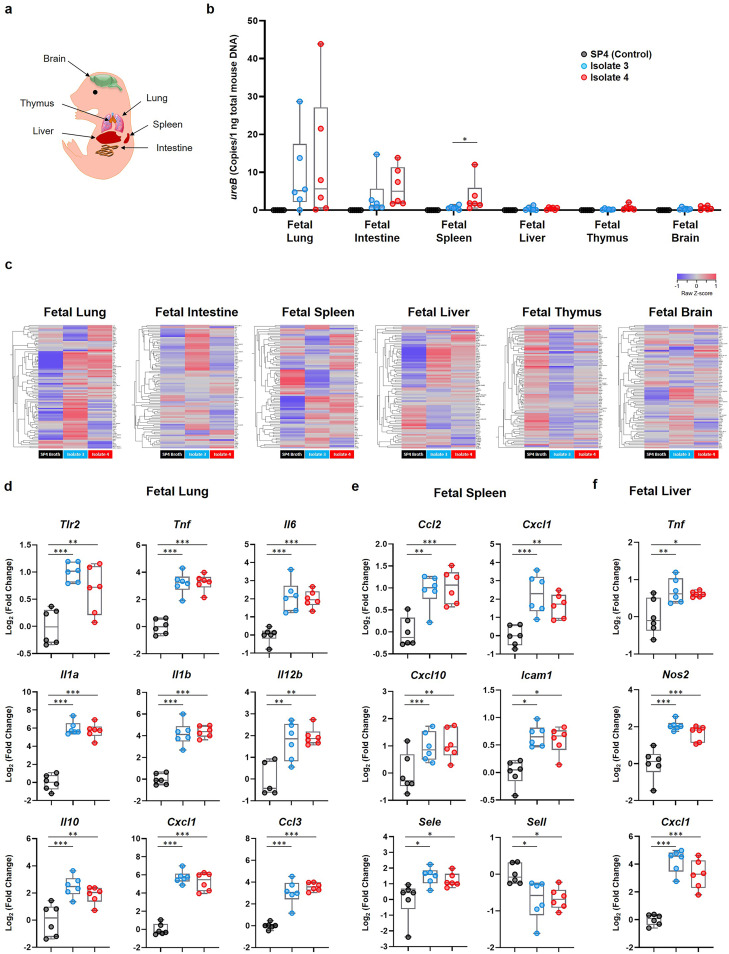
Bacterial burden and inflammatory gene expression in the fetal organs upon inoculation with *U. parvum* serovar 14. Dams were intra-amniotically inoculated with *U. parvum* isolate 3 (1.0 × 10^4^ cells/25 μl) or *U. parvum* isolate 4 (1.0 × 10^4^ cells/25 μl), or injected with vehicle control (SP4 broth, 25 μl) at 16.5 days *post coitum* (dpc) (*n* = 6 each). Tissues were collected 16 h after intra-amniotic inoculation. (a) Spatial localization of the murine fetal organs included in this experiment. (b) Copy numbers of the *ureB* gene in the fetal organs. (c) Heatmap visualization of inflammatory gene expression in the fetal organs. (d to f) Expression of specific inflammatory genes in the fetal lung (d), spleen (e), and liver (f). The data are shown as box-and-whisker plots, where midlines indicate medians, boxes indicate interquartile ranges, and whiskers indicate minimum and maximum ranges. The *P* values were determined by Mann-Whitney U tests (b) or one-way analysis of variance (ANOVA), followed by Tukey’s tests to determine differences between groups (d to f). *, *P* < 0.05; **, *P* < 0.01; ***, *P* < 0.001.

To further explore the effects of *U. parvum* serovar 14 on the fetus, we first evaluated whether isolate 3 and isolate 4 can be detected in the fetal organs. The fetal lung, intestine, spleen, liver, thymus, and brain were collected from pregnant dams intra-amniotically inoculated with *U. parvum* isolate 3 or isolate 4 (Fig. 4a), and the bacterial burden was quantified by real-time quantitative PCR (qPCR) of the *ureB* gene. Both isolates of *U. parvum* were most abundant in the fetal lung, followed by the fetal intestine and fetal spleen (Fig. 4b). Interestingly, the abundance of isolate 4 in the fetal spleen was higher than that of isolate 3 (Fig. 4b). There was a detectable yet minimal bacterial burden in the fetal liver, thymus, and brain (Fig. 4b). Fetal organs from PBS- and vehicle control-injected dams had no detectable *ureB* gene copies (Fig. 4b; see also Fig. S4a in the supplemental material). These results show that when *U. parvum* serovar 14 invades the amniotic cavity, the primary sites of infection are the fetal lung, fetal intestine, and fetal spleen.

10.1128/mBio.00797-20.4 FIG S4 Bacterial burden in the fetal, maternal, reproductive, and gestational tissues from dams intra-amniotically injected with PBS. (a) Copy numbers of the *ureB* gene in the fetal organs. (b) Copy numbers of the *ureB* gene in the maternal, reproductive, and gestational tissues. Download FIG S4, TIF file, 0.1 MB. Copyright © 2020 Motomura et al. 2020 Motomura et al. This content is distributed under the terms of the Creative Commons Attribution 4.0 International license.

Next, we evaluated the expression of several inflammatory mediators in the fetal organs upon infection with *U. parvum* serovar 14. Intra-amniotic inoculation with either isolate of *U. parvum* serovar 14 induced the upregulation of multiple inflammatory genes in the fetal lung, spleen, and liver, with the number of upregulated genes in the fetal lung being highest among the fetal tissues (Fig. 4c). Specifically, the expression of *Tlr2*, *Tnf*, *Il6*, *Il1a*, *Il1b*, *Il12b*, *Il10*, *Cxcl1*, and *Ccl3* was significantly upregulated in the fetal lung from dams intra-amniotically inoculated with *U. parvum* serovar 14 (Fig. 4d). The fetal intestine contained the second greatest bacterial burden among the fetal organs (Fig. 4b); however, only the expression of *Il1b* was significantly upregulated in this fetal organ from dams inoculated with either *U. parvum* isolate (Fig. S5a). In the fetal spleen, the relative expression of *Ccl2*, *Cxcl1*, *Cxcl10*, *Icam1*, *Sele*, and *Sell* was significantly altered in response to either *U. parvum* isolate (Fig. 4e). Although the bacterial burden of the fetal liver was low (Fig. 4b), the relative expression of *Tnf*, *Nos2*, and *Cxcl1* was upregulated in response to isolate 3 or isolate 4 (Fig. 4f). No differences in the expression of the above-mentioned inflammatory genes were observed between fetal organs from PBS- or vehicle control-injected dams (Fig. S5b and S6a to c). Together, these findings indicate that a primary site of *U. parvum* serovar 14 invasion in the fetus is the lung, in which a significant bacterial burden and upregulation of inflammatory mediators take place.

10.1128/mBio.00797-20.5 FIG S5 Inflammatory gene expression in the fetal intestine from dams intra-amniotically inoculated with U. parvum or injected with SP4 broth or PBS. (a) Expression of *Il1b* in the fetal intestine from dams intra-amniotically inoculated with *U. parvum* serovar 14 isolate 3 or *U. parvum* serovar 14 isolate 4, or injected with SP4 broth (control). (b) Expression of *Il1b* in the fetal intestine from dams intra-amniotically injected with PBS or SP4 broth. The data are shown as box-and-whisker plots, where midlines indicate medians, boxes indicate interquartile ranges, and whiskers indicate minimum and maximum ranges. Statistical analysis was performed using one-way analysis of variance (ANOVA), followed by a Tukey’s test, to determine differences between groups (a), or an unpaired *t* test (b). **, *P* < 0.01; ***, *P* < 0.001. Download FIG S5, TIF file, 0.04 MB. Copyright © 2020 Motomura et al. 2020 Motomura et al. This content is distributed under the terms of the Creative Commons Attribution 4.0 International license.

10.1128/mBio.00797-20.6 FIG S6 Inflammatory gene expression in the fetal organs from dams intra-amniotically injected with PBS or SP4 broth. (a to c) Expression of specific inflammatory genes in the fetal lung (a), fetal spleen (b), and fetal liver (c) from dams intra-amniotically injected with PBS or SP4 broth. The data are shown as box-and-whisker plots, where midlines indicate medians, boxes indicate interquartile ranges, and whiskers indicate minimum and maximum ranges. Statistical analysis was performed by using unpaired *t* tests. The results of all statistical analyses were non-significant. Download FIG S6, TIF file, 0.1 MB. Copyright © 2020 Motomura et al. 2020 Motomura et al. This content is distributed under the terms of the Creative Commons Attribution 4.0 International license.

### *U. parvum* serovar 14 is detected in the reproductive tissues and induces a local inflammatory response.

**FIG 5:**
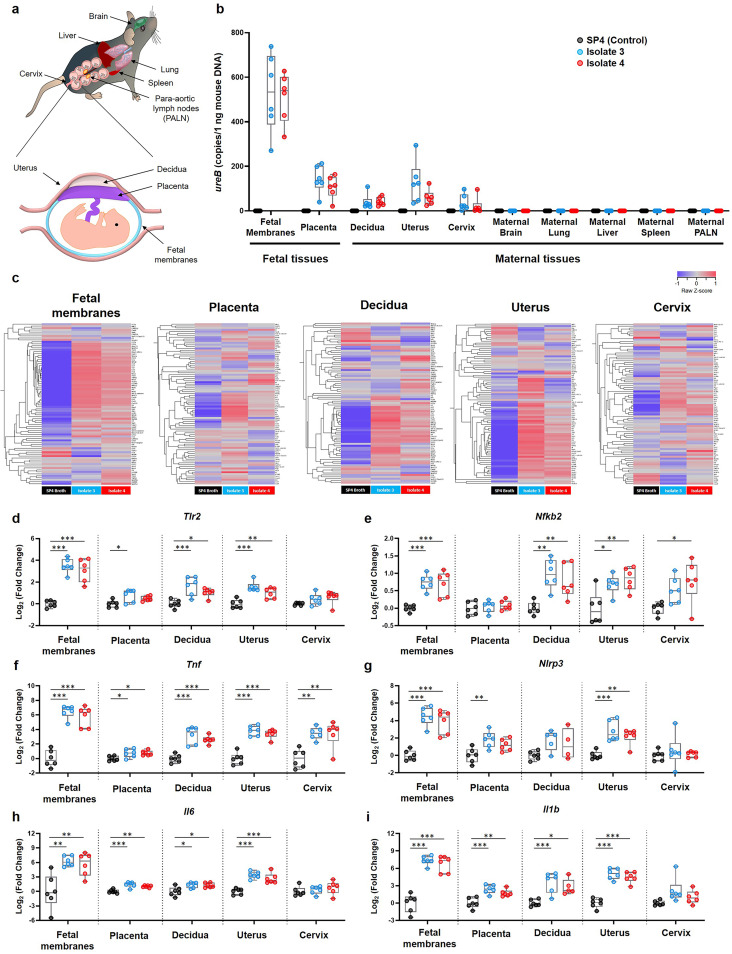
Bacterial burden and inflammatory gene expression in reproductive and gestational tissues upon inoculation with *U. parvum* serovar 14. Dams were intra-amniotically inoculated with *U. parvum* isolate 3 (1.0 × 10^4^ cells/25 μl) or *U. parvum* isolate 4 (1.0 × 10^4^ cells/25 μl), or injected with vehicle control (SP4 broth, 25 μl) at 16.5 days *post coitum* (dpc) (*n* = 6 each). Tissues were collected 16 h after intra-amniotic inoculation. (a) Spatial localization of the murine tissues collected in this experiment. (b) Copy numbers of the *ureB* gene in maternal, fetal, and reproductive tissues. (c) Heatmap visualization of inflammatory gene expression in the fetal membranes, placenta, decidua, uterus, and cervix. (d to i) Expression of *Tlr2* (d), *Nfkb2* (e), *Tnf* (f), *Nlrp3* (g), *Il6* (h), and *Il1b* (i) in the fetal membranes, placenta, decidua, uterus, and cervix. The data are shown as box-and-whisker plots, where midlines indicate medians, boxes indicate interquartile ranges, and whiskers indicate minimum and maximum ranges. The *P* values were determined by Mann-Whitney U tests (b) or one-way analysis of variance (ANOVA), followed by Tukey’s tests to determine differences between groups (d to i). *, *P* < 0.05; **, *P* < 0.01; ***, *P* < 0.001.

Similar to the fetus, microbes in the amniotic cavity can also invade the surrounding tissues such as the chorioamniotic membranes (also known as fetal membranes) and placenta (31, 32). Thus, we proceeded to evaluate whether intra-amniotic *U. parvum* serovar 14 can be detected in the reproductive tissues surrounding the amniotic cavity as well as the maternal organs. The fetal membranes, placenta, decidua, uterus, cervix, maternal brain, maternal lung, maternal liver, maternal spleen, and the maternal para-aortic lymph nodes (PALN) were collected from pregnant dams intra-amniotically inoculated with *U. parvum* isolate 3 or isolate 4 (Fig. 5a), and the bacterial burden was quantified by qPCR of the *ureB* gene. The greatest bacterial burden was found in the fetal membranes, distantly followed by the placenta, decidua, uterus, and cervix compared to vehicle controls (Fig. 5b). There was no detectable bacterial burden in the maternal brain, lung, liver, spleen, and PALN (Fig. 5b). Reproductive and maternal organs from PBS- and vehicle control-injected dams had no detectable *ureB* gene copies (Fig. 5b; see also Fig. S4b in the supplemental material). These results show that intra-amniotic *U. parvum* serovar 14 disseminates into the surrounding tissues (e.g., fetal membranes and placenta) and also reaches the maternal-fetal interface (e.g., decidua) and reproductive tissues (e.g., uterus and cervix).

U. parvum serovar 14 was detected only in the gestational and reproductive tissues; therefore, we next evaluated the expression of inflammatory mediators in these compartments. The fetal membranes, placenta, decidua, uterus, and cervix each displayed consistent upregulation of inflammatory mediators upon intra-amniotic infection with isolate 3 or isolate 4 compared to vehicle controls (Fig. 5c). Interestingly, the fetal membranes and uterus had the greatest upregulation of inflammatory genes, whereas the placenta displayed a weaker response (Fig. 5c).

We then compared the relative expression of specific inflammatory genes (*Tlr2*, *Nfkb2*, *Tnf*, *Nlrp3*, *Il6*, and *Il1b*) in the gestational and reproductive tissues upon inoculation with *U. parvum* isolate 3 or isolate 4. Strikingly, the fetal membranes displayed the highest expression of inflammatory genes (Fig. 5d and f to i), which corresponded to the high bacterial burden detected in these tissues (Fig. 5b). Specifically, the expression of *Tlr2* was upregulated in the fetal membranes, decidua, uterus, and—to a lesser degree—in the placenta in response to both isolates (Fig. 5d). The expression of the transcription factor *Nfkb2* was upregulated in every tissue, except for the placenta, in response to both isolates (Fig. 5e). The expression of *Tnf* was upregulated in all tissues in response to both isolates (Fig. 5f), whereas the expression of *Nlrp3* (the sensor molecule of the NLRP3 inflammasome [33]) was upregulated only in the fetal membranes, placenta, and uterus (Fig. 5g). Consistently, the expression of *Il6* and *Il1b* increased in response to both isolates in the fetal membranes, placenta, decidua, and uterus but not in the cervix (Fig. 5h and i). The expression of *Tlr2*, *Nfkb2*, *Tnf*, *Nlrp3*, *Il6*, and *Il1b* in PBS- and vehicle control-injected dams is shown in Fig. S7. These results show that intra-amniotic inoculation with *U. parvum* serovar 14 results in the upregulation of several inflammatory mediators in the gestational and reproductive tissues.

10.1128/mBio.00797-20.7 FIG S7 Inflammatory gene expression in the reproductive and gestational tissues from dams intra-amniotically injected with PBS or SP4 broth. The expression of specific inflammatory genes in the fetal membranes, placenta, decidua, uterus, and cervix of dams intra-amniotically injected with PBS or SP4 broth. The data are shown as box-and-whisker plots where midlines indicate medians, boxes indicate interquartile ranges, and whiskers indicate minimum and maximum ranges. *P* values were determined by using unpaired *t* tests. *, *P* < 0.05. Download FIG S7, TIF file, 0.1 MB. Copyright © 2020 Motomura et al. 2020 Motomura et al. This content is distributed under the terms of the Creative Commons Attribution 4.0 International license.

### Does *U. parvum* serovar 14 induce the upregulation of inflammatory mediators in human amniocytes *in vitro*?

**FIG 6:**
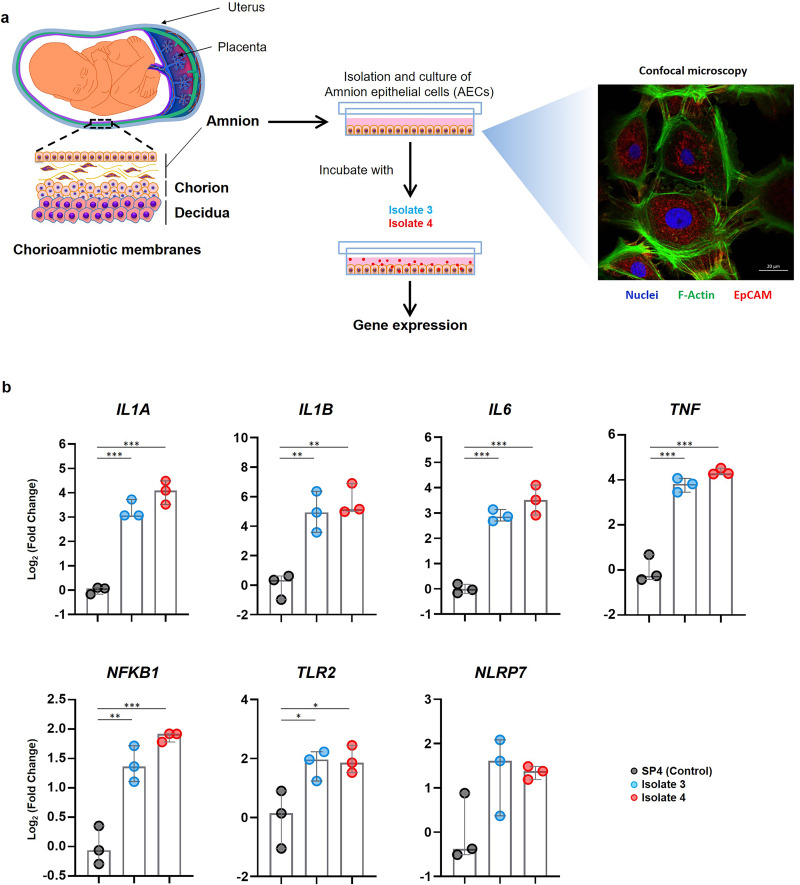
Incubation of U. parvum serovar 14 and primary human amniocytes. (a) Experimental design (left to right). Primary human amnion epithelial cells (AECs) (*n* = 3) were isolated from term human chorioamniotic membranes and incubated with *U. parvum* isolate 3 (1.3 × 10^5^ cells), *U. parvum* isolate 4 (1.3 × 10^5^ cells), or vehicle control (SP4 broth) for 24 h. The AECs were then collected to determine gene expression. A representative confocal microscopy image of the immunofluorescence staining of AECs to show the expression of EpCAM and F-Actin is displayed. (b) Expression of *IL1A*, *IL1B*, *IL6*, *TNF*, *NFKB1*, *TLR2*, and *NLRP7* in AECs incubated with *U. parvum* isolates. The data are shown as medians with minimum and maximum ranges. The *P* values were determined by one-way analysis of variance (ANOVA), followed by Tukey’s tests to determine differences between groups. *, *P* < 0.05; **, *P* < 0.01; ***, *P* < 0.001.

Given that the fetal membranes were the primary site of *U. parvum* serovar 14 infection resulting in the upregulation of inflammatory mediators, we investigated whether this bacterium could induce similar immune responses in the inner layer (i.e., amnion epithelial cells [AECs]) of the chorioamniotic membranes, which faces the amniotic cavity. AECs were isolated from the placentas of normal term deliveries and incubated with *U. parvum* isolate 3 or isolate 4 (Fig. 6a). A representative confocal microscopy image of primary cultured AECs is shown in Fig. 6a. Consistent with our murine findings (Fig. 5c to i), AECs incubated with either of the *U. parvum* serovar 14 isolates had significantly higher relative expression of the inflammatory genes *IL1A*, *IL1B*, *IL6*, *TNF*, *NFKB1*, and *TLR2* compared to the vehicle control (Fig. 6b). The expression of *NLRP7*, an inflammasome sensor molecule upregulated upon infection with *Ureaplasma* species (34), tended to increase as well, although this did not reach statistical significance (Fig. 6b). The expression of *NLRP3* was not upregulated in response to either *U. parvum* isolate (Fig. S8a). The expression of *IL1A*, *IL1B*, *IL6*, *TNF*, *NFKB1*, *TLR2*, *NLRP7*, and *NLRP3* in AECs incubated with PBS or vehicle control is shown in Fig. S8b. These data indicate that *U. parvum* serovar 14 upregulates the expression of several inflammatory mediators in human amniocytes, resembling the inflammatory milieu observed in the fetal membranes of animals intra-amniotically inoculated with this bacterium.

10.1128/mBio.00797-20.8 FIG S8 Expression of specific inflammatory genes in primary human amniocytes incubated with U. parvum serovar 14, PBS, or SP4 broth. (a) Expression of *NLRP3* in primary human amnion epithelial cells incubated with *U. parvum* isolate 3, isolate 4, or SP4 broth. (b) Expression of inflammatory genes in primary human amnion epithelial cells incubated with PBS or SP4 broth. The data are shown as medians with minimum and maximum ranges. *P* values were determined by one-way analysis of variance (ANOVA), followed by a Tukey’s test, to determine differences between groups (a), or unpaired *t* tests (b). **, *P* < 0.01; ***, *P* < 0.001. Download FIG S8, TIF file, 0.1 MB. Copyright © 2020 Motomura et al. 2020 Motomura et al. This content is distributed under the terms of the Creative Commons Attribution 4.0 International license.

### Treatment with clarithromycin reduces adverse pregnancy and neonatal outcomes induced by *U. parvum* serovar 14.

**FIG 7:**
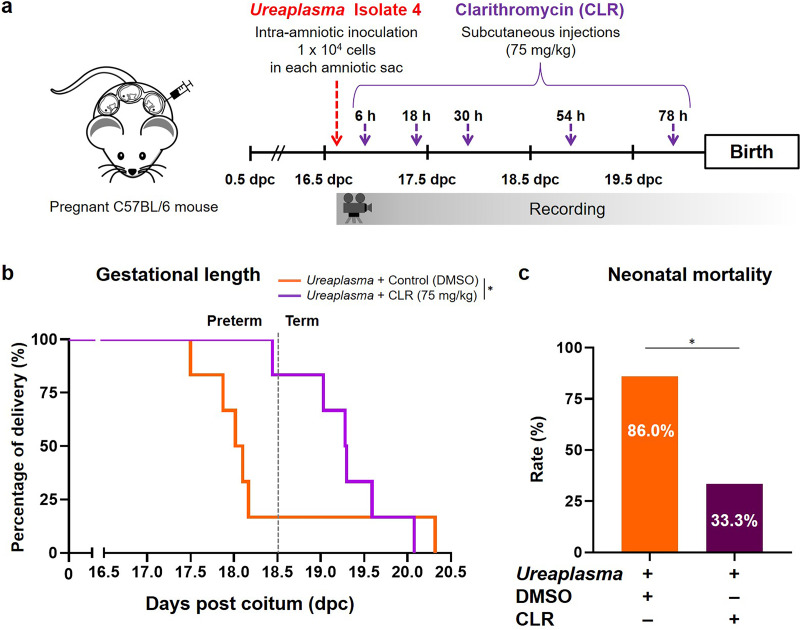
Clarithromycin treatment after intra-amniotic inoculation with U. parvum serovar 14. (a) Experimental design. Dams were intra-amniotically inoculated with *U. parvum* isolate 4 (1.0 × 10^4^ cells/25 μl) and subcutaneously treated with clarithromycin (CLR; 75 mg/kg) or vehicle control (DMSO) at 6, 18, 30, 54, and 78 h post-inoculation and monitored until delivery (*n* = 6 each). (b) Kaplan-Meier survival curves showing the gestational lengths at delivery. The *P* value was determined by a Gehan-Breslow-Wilcoxon test. (c) Neonatal mortality. The *P* value was determined by an unpaired *t* test. *, *P* < 0.05.

Recent clinical studies have shown that the administration of antibiotics improves perinatal outcomes in women with intra-amniotic infection/inflammation (22–24). Three antibiotics were administered in these studies to achieve broad coverage, but among these three only clarithromycin (CLR) is effective against *Ureaplasma* species (35). Therefore, we tested whether treatment with this antibiotic could prevent adverse pregnancy and neonatal outcomes in dams intra-amniotically inoculated with *U. parvum* serovar 14 (Fig. 7a). We verified the efficacy of CLR against intra-amniotic infection with isolate 4 (low dose), since this isolate of *U. parvum* serovar 14 caused the highest rate of preterm birth as well as a significant rate of neonatal mortality (Fig. 2). The gestational length of dams intra-amniotically inoculated with *U. parvum* serovar 14 and treated with CLR was significantly longer than those treated with vehicle control, resulting in a reduced rate of preterm birth (Fig. 7b). Moreover, treatment with CLR significantly reduced neonatal mortality in dams intra-amniotically inoculated with *U. parvum* serovar 14 (Fig. 7c). These results demonstrate that the adverse pregnancy and neonatal consequences caused by intra-amniotic infection with *U. parvum* serovar 14 can be ameliorated using clarithromycin, a recently recommended antibiotic.

## DISCUSSION

The present study provides evidence of a causal link between clinically isolated *Ureaplasma* species and adverse pregnancy and neonatal outcomes. First, taxonomic characterization of four *Ureaplasma* isolates from women with intra-amniotic infection revealed that the most common *Ureaplasma* species in this clinical condition is *U. parvum*. However, no differences in known genetic virulence factors were found among clinically obtained *Ureaplasma* isolates. Therefore, the bacterial virulence cannot be the sole factor accounting for disease severity among the four included patients, suggesting that host-pathogen interactions may dictate the pregnancy outcome. Next, using animal models, we showed that intra-amniotic inoculation with clinically obtained *Ureaplasma* isolates induces preterm birth and neonatal mortality to variable degrees. Further, we demonstrated that *U. parvum* serovar 14 induces a severe intra-amniotic inflammatory response in the absence of a maternal systemic inflammatory response. Importantly, *U. parvum* serovar 14 invaded the fetal lung, intestine, and spleen, inducing fetal inflammatory response syndrome. This bacterium was also found in the fetal membranes, placenta, and decidua as well as the reproductive tissues, inducing severe inflammatory responses primarily in the fetal membranes. *In vitro* studies showed the relevance of our *in vivo* findings by demonstrating that inoculation with *U. parvum* serovar 14 triggers proinflammatory responses in human amniocytes. Lastly, we demonstrated the efficacy of clarithromycin, a recently recommended yet not widely utilized antibiotic, in abrogating the adverse pregnancy and neonatal outcomes induced by *Ureaplasma* species.

Initially, we identified taxonomic differences among the four clinically obtained *Ureaplasma* isolates, indicating that there is diversity in the *Ureaplasma* species that invade the amniotic cavity. However, the broad genomic analysis performed here did not allow us to identify differences in known virulence factors among *Ureaplasma* isolates obtained from women with intra-amniotic infection. Possible explanations for why the difference in these genomic factors is unable to distinguish the virulence of *Ureaplasma* species in the context of preterm delivery include the following: (i) unknown strain-specific virulence factors exist and affect the pregnancy outcomes; (ii) the inflammation induced by *Ureaplasma* species not only depends on this bacterium but also on host factors such as genetic predisposition (36); or (iii) intra-amniotic infection with *Ureaplasma* species slightly alters the timing of birth in all humans/animals in a manner that was imperceptible in the present study. In addition, the number of *Ureaplasma* isolates included in this study may have limited our capacity to detect subtle differences between strains. A deeper whole-genome analysis is required to identify novel strain-specific virulence factors that may dictate pregnancy disease. In the present study, we focused on the *in vivo* effects of *Ureaplasma* species invading the amniotic cavity.

Intra-amniotic inoculation with clinically isolated *Ureaplasma* species caused preterm birth and neonatal mortality that varied between isolates. Importantly, *U. parvum* serovar 14 obtained from a woman who delivered preterm caused the highest rates of preterm birth and neonatal mortality, indicating that this specific isolate displayed an intrinsically greater degree of virulence than other isolates. To our knowledge, this is the first demonstration that clinically isolated *U. parvum* serovar 14 has been shown to induce preterm birth in an animal model. In our study, we also found that intra-amniotic inoculation with *U. parvum* serovar 3 induced a low rate of preterm birth but high rates of neonatal mortality. This finding contrasts with a previous report showing that intra-amniotic inoculation with *U. parvum* serovar 3 via mini-laparotomy did not induce preterm birth (21). The variability in the rates of preterm birth and neonatal mortality caused by the different *Ureaplasma* isolates indicates that individual host susceptibility to intra-amniotic infection contributes to the severity of adverse pregnancy outcomes.

In humans, *Ureaplasma*-associated intra-amniotic infection causes a local inflammatory response in the absence of maternal systemic inflammation, preventing noninvasive diagnosis of this clinical condition (37). This finding is consistent with the present study, in which *Ureaplasma* inoculation neither induced a maternal inflammatory response nor were *Ureaplasma* strains detected in systemic maternal organs such as the brain, lung, liver, and lymphatic organs. On the other hand, intra-amniotic inoculation with *Ureaplasma* isolates induced high rates of neonatal mortality, which coincided with intra-amniotic and fetal inflammatory responses. Importantly, intra-amniotic inoculation with *Ureaplasma* species induced a surge of proinflammatory cytokines (e.g., IL-6 and CXCL10), which are conventionally measured for the diagnosis of intra-amniotic inflammation (28, 38). However, this intra-amniotic cytokine storm alone is insufficient to cause fetal disease; therefore, we also investigated the microbial burden and the inflammatory response in the fetus. Consistent with human research in the fetal inflammatory response syndrome (a clinical condition resulting from fetal exposure to microbial invasion of the amniotic cavity [39, 40]), the fetuses of dams intra-amniotically inoculated with *U. parvum* displayed severe inflammatory responses primarily in the lung, spleen, and liver. The most affected fetal organ was the lung, which also contained the highest microbial burden. These observations are supported by previous reports showing that fetal exposure to *Ureaplasma* species induces an increased expression of proinflammatory cytokines in the fetal lung of mice (21) as well as an influx of immune cells in that of rhesus macaques (19). Fetal lung inflammation characterized by immune cell influx and elevated cytokine expression is also observed after chronic *Ureaplasma* infection in the amniotic cavity in ovine models (41). Moreover, clinical studies have described that prenatal exposure to *Ureaplasma* species is associated with chronic lung disease (42) or bronchopulmonary dysplasia (43, 44). The association of *Ureaplasma* species with fetal lung complications observed in multiple animal models and in humans may be explained by the fact that the fetal lung is directly exposed to bacteria in the amniotic cavity, as the fetus can aspirate amniotic fluid (45). In the present study, we also found that *Ureaplasma* species were detected in the fetal intestine, which is in tandem with a clinical demonstration showing that fetuses exposed to these bacteria can develop necrotizing enterocolitis in the neonatal period (46). Therefore, it is likely that the intense inflammatory processes induced by *Ureaplasma* species in mice can subsequently lead to organ damage, as observed in humans (47), which may explain the high rate of neonatal mortality.

U. parvum was not only detected in the fetal organs but also in the gestational tissues and at the maternal-fetal interface (i.e., the decidua). These observations coincide with clinical studies showing the presence of bacteria in the placenta (32), chorioamniotic membranes (31), and choriodecidua (48) in women with intra-amniotic infection. The microbial invasion of these tissues is strongly associated with a proinflammatory milieu (49), as observed in this study. However, we found that, among the fetal and maternal tissues, *U. parvum* invasion was greatest in the fetal membranes and coincided with a severe inflammatory response, which is consistent with recent findings showing that vaginal inoculation with *U. parvum* results in an ascending infection that reaches the fetal membranes, inducing local inflammation (50). Interestingly, the immune response induced by inoculation with *U. parvum* involves the activation of the NLRP3 inflammasome, an important pathway for preterm parturition in the context of intra-amniotic infection (51–53). Of note, our *in vitro* studies showed that the incubation of primary amniocytes with *U. parvum* also induced an inflammatory response in the absence of the upregulation of the NLRP3 transcript, suggesting that the choriodecidua is central in the intra-amniotic activation of the inflammasome in humans.

It is worth mentioning that intra-amniotic inoculation with *U. parvum* also resulted in the detection of this bacterium in the uterus and cervix. There are two plausible explanations for this finding: (i) the intra-amniotic infection progressed beyond the amniotic cavity into the reproductive tissues, and/or (ii) inoculation with *U. parvum*, or the bacterium itself, induced rupture of the fetal membranes, thus facilitating the spread of infection. Regardless, the presence of *U. parvum* in the uterus and cervix resulted in the upregulation of several inflammatory mediators that, in turn, led to the activation of the common pathway of parturition (5).

Here, we are the first to demonstrate that clarithromycin abrogates *Ureaplasma-*induced preterm birth and neonatal mortality. Notably, recent studies have shown that clarithromycin can be used together with ceftriaxone and metronidazole to treat preterm birth in women with intra-amniotic infection (22–24). Clarithromycin is a macrolide antibiotic, which acts against *Mollicutes* (including *Ureaplasma* species) by inhibiting bacterial protein synthesis (54). In addition to the bacteriostatic function against *Ureaplasma* species, this antibiotic exhibits immunomodulatory effects by suppressing the NF-κB pathway (55), which was shown in this and other studies to be activated by *Ureaplasma* species (56). Together, these results provide mechanistic evidence that the recent recommendations to use clarithromycin in the treatment of women with intra-amniotic infection (22–24) are substantiated in clinical practice.

**FIG 8:**
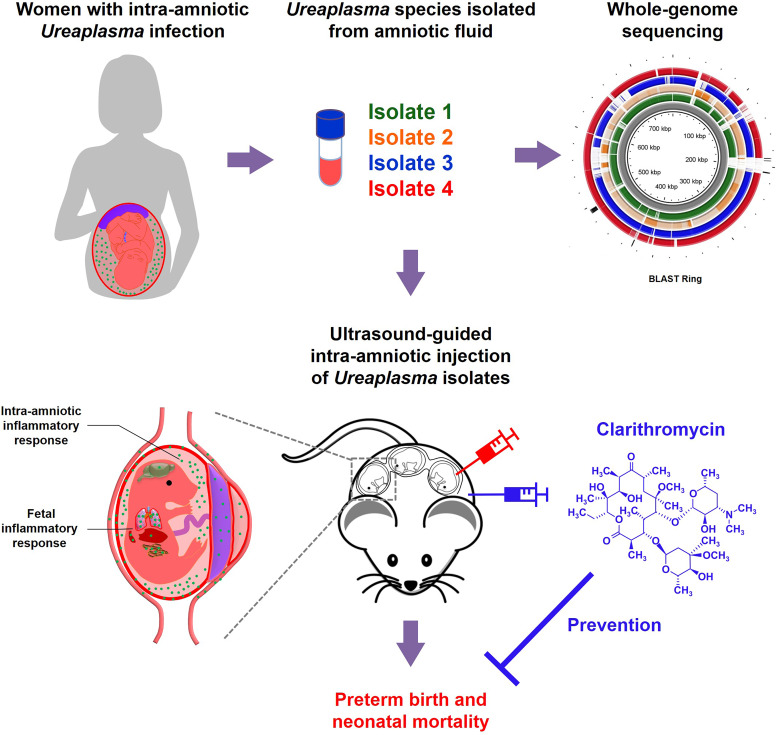
Graphical abstract. *Ureaplasma* species were isolated from amniotic fluid of four women with intra-amniotic *Ureaplasma* infection. Whole-genome sequencing of each *Ureaplasma* isolate was performed (BLAST Ring shown in Fig. 1), and the isolates were taxonomically characterized, indicating that U. parvum was the most common genital mycoplasma in the amniotic cavity. The ultrasound-guided intra-amniotic injection of *Ureaplasma* species into pregnant mice resulted in adverse pregnancy and neonatal outcomes driven by severe intra-amniotic and fetal inflammatory responses, as well as the invasion of *Ureaplasma* species into specific tissues. Importantly, treatment with clarithromycin successfully prevented *Ureaplasma*-induced adverse pregnancy and neonatal outcomes.

In summary, the present study provides a taxonomic characterization of *Ureaplasma* species found in women with intra-amniotic infection. In addition, mechanistic experimentation shows that *U. parvum* induces adverse pregnancy and neonatal outcomes by causing a severe inflammatory response in the amniotic cavity, the fetus, the gestational and reproductive tissues, and the maternal-fetal interface. Importantly, our data provide solid evidence demonstrating that treatment with clarithromycin, an antibiotic that is not yet widely utilized, must be implemented in the management of women with intra-amniotic infection, given that *Ureaplasma* species are the most commonly detected microbes in such patients (Fig. 8). Collectively, our investigations provide insights into the maternal-fetal immunobiology of intra-amniotic infection, the most established link to prematurity and neonatal morbidity and mortality worldwide.

## MATERIALS AND METHODS

### Ethics statement. (i) Human subjects and clinical specimens.

Human amniotic fluid samples and chorioamniotic membranes were obtained at the Perinatology Research Branch, an intramural program of the *Eunice Kennedy Shriver* National Institute of Child Health and Human Development, National Institutes of Health, U.S. Department of Health and Human Services, Wayne State University (Detroit, MI), and the Detroit Medical Center (Detroit, MI). The collection and use of human materials for research purposes were approved by the Institutional Review Boards of the National Institute of Child Health and Human Development and Wayne State University. All participating women provided written informed consent prior to sample collection.

### (ii) Mice.

Animal procedures were approved by the Institutional Animal Care and Use Committee (IACUC) at Wayne State University (protocols A 07-03-15 and 18-03-0584). We adhered to the National Institutes of Health *Guide for the Care and Use of Laboratory Animals*.

### Amniotic fluid sample collection.

Amniotic fluid was retrieved by transabdominal amniocentesis under antiseptic conditions using ultrasound monitoring from women who were suspected to have intra-amniotic inflammation/infection (see clinical definitions below). All samples were collected to assess the presence of intra-amniotic infection (see clinical definitions below). The clinical and demographic characteristics of the study population are shown in Table S1 in the supplemental material. Amniotic fluid samples were transported to the clinical laboratory in a capped sterile syringe to perform research cultures for *Ureaplasma* species. In addition, an aliquot of amniotic fluid was transported to the clinical laboratory for culture of aerobic/anaerobic bacteria and genital mycoplasmas, as previously described (1, 7). The clinical and research tests also included the determination of an amniotic fluid white blood cell count, Gram stain examination, glucose concentration, and IL-6 concentration, as previously performed (57).

### Clinical definitions and placental histopathologic examination.

The presence of viable bacteria in the amniotic cavity was evaluated by amniotic fluid culture as previously described and included culture for genital mycoplasmas. Intra-amniotic inflammation was defined as an amniotic fluid IL-6 concentration of ≥2.6 ng/ml (28). Microbial-associated intra-amniotic inflammation, or intra-amniotic infection, was defined as the presence of cultivable bacteria together with intra-amniotic inflammation (57). Histopathological examination of the placenta was performed by perinatal pathologists blinded to clinical diagnoses and obstetrical outcomes according to standardized Perinatology Research Branch protocols (58). Acute inflammatory lesions of the placenta (maternal inflammatory response and fetal inflammatory response) were diagnosed according to established criteria, including staging and grading (58). Preterm delivery was defined as delivery <37 weeks of gestation whereas term delivery occurred after 37 weeks of gestation.

### Isolation of *Ureaplasma* species from amniotic fluid.

Amniotic fluid samples were inoculated in SP4 broth with urea (catalog no. R87; Hardy Diagnostics, Santa Maria, CA) to selectively culture *Ureaplasma* species. A change in the pH of the media resulted in a color change from yellow to red, which visually indicated the growth of bacteria. The color-changed media (*n* = 4) was mixed with 50% glycerol (Teknova, Hollister, CA; with a final concentration of glycerol of 15 to 25%), aliquoted, and stored at –80°C (original stocks, isolates 1 to 4). These isolates were used for *in vivo* and *in vitro* experiments. The clinical characteristics of these four patients are shown in Table S1.

### Isolation of DNA from *Ureaplasma* isolates.

Amniotic fluid *Ureaplasma* isolates were recovered from original stocks (isolates 1 to 4) in SP4 broth with urea. Bacterial cells were pelleted by centrifugation at 10,000 × *g* at 4°C for 20 min. DNA was extracted from the pellets of bacterial cells using the DNeasy PowerSoil kit (Qiagen, Hilden, Germany) with minor modifications to the manufacturer’s protocol. Specifically, the pellets were resuspended in 200 μl of the bead solution, and the suspension was returned to the bead tube. Bacterial cells were lysed by mechanical disruption for 30 s using a bead beater (Mini-Beadbeater 16; Biospec Products, Bartlesville, OK). After centrifugation, the supernatants were transferred to new tubes, 100 μl of solution C2 and 100 μl of solution C3 were added, and the tubes were incubated at 4°C for 5 min. Finally, 60 μl of solution C6 was placed on the filter columns and incubated for 5 min before centrifuging for 30 s to elute the extracted DNA. Purified DNA was stored at –20°C.

### Taxonomic identification of *Ureaplasma* isolates.

The 16S rRNA gene of each *Ureaplasma* isolate was amplified using the 27F/1492R primer set (5′-AGAGTTTGATCMTGGCTCAG-3′ / 5′-TACCTTGTTACGACTT-3′) and then bidirectionally Sanger sequenced by Genewiz (South Plainfield, NJ) using the 515F/806R primer set (5′-GTGYCAGCMGCCGCGGTAA-3′ / 5′-GGACTACNVGGGTWTCTAAT-3′), which targets the V4 hypervariable region of the 16S rRNA gene. Forward and reverse reads were trimmed using Chromatogram Explorer Lite (Heracle BioSoft S.R.L, Arges, Romania) with default settings; the reads were then assembled using the CAP (contig assembly program) of BioEdit software (v7.0.5.3), also with default settings. The taxonomic identities of individual bacterial isolates were determined using the Basic Local Alignment Search Tool (BLAST) (59). If a matching bacterial strain in the nucleotide collection (nr/nt) database had more than a single copy of the 16S rRNA gene, the percent nucleotide similarity values between the isolate and the strain were reported for the best matching sequence.

### Genome sequencing, assembly, and analysis.

Amniotic fluid *Ureaplasma* isolates were recovered from original stocks in SP4 broth with urea. Bacterial cells were pelleted by centrifugation at 3,000 × *g* at room temperature for 30 min. The cells were washed with Tris-EDTA (TE) buffer (Teknova), resuspended in TE buffer, stored at –80°C, and sent to CosmosID (Rockville, MD) for DNA isolation, genomic sequencing, contig assembly, SNP analysis, phylogenetic analysis, antibiotic resistance analysis, and virulence factor analysis. Raw sequence reads were trimmed through the BBduk (BBMap package) package tool (60) using minlen = 25, and contig sequences were assembled in SPAdes (61) using 77, 99, and 127 k-mer sizes. Coding sequences were then annotated using the Prokka pipeline (62). For identification of antibiotic resistance genes and virulence factor genes, contigs were compared against CosmosID’s in-house databases. Antibiotic resistance and virulence factor genes were considered to be present if their sequences matched with those in an assembled genome at a >90% identity with 60% coverage. Phylograms were constructed from the alignment of the SNPs extracted for each isolate using Harvest Suite (including Parsnp and Gingr) (63).

Secondarily, contigs were assembled from raw sequence reads using CLC Genomics Workbench version 12 (Qiagen, Valencia, CA). Contig assembly settings included mapping reads back to the assembled contigs, updating contigs, automatic word and bubble sizes, and limiting the minimum contig length to 200 bp. Genome scaffolds were then generated by mapping contigs to a reference genome, either ATCC 27815 (CP000942) for *U. parvum* or ATCC 33699 (CP001184) for *U. urealyticum*. Multiple genome alignment was then performed using the BLAST Ring Image Generator (BRIG) (64) to compare whole-genome sequence similarities.

### Mice.

C57BL/6 mice were purchased from The Jackson Laboratory (Bar Harbor, ME) and bred in the animal care facility at the C. S. Mott Center for Human Growth and Development at Wayne State University (Detroit, MI). All mice were kept under a circadian cycle (light:dark, 12:12 h). Females, 8 to 12 weeks old, were bred with males of proven fertility. Female mice were checked daily between 8:00 a.m. and 9:00 a.m. for the appearance of a vaginal plug, which indicated 0.5 days *post coitum* (dpc). Females were then housed separately from the males, their weights were monitored daily, and a gain of two or more grams by 12.5 dpc confirmed pregnancy.

### Preparation of *Ureaplasma* isolates for intra-amniotic inoculation.

The original stocks of the four clinical *Ureaplasma* isolates (isolates 1 to 4) were inoculated in SP4 broth and cultured at 37°C for 18 to 24 h. The culture medium was then mixed with the same volume of 50% glycerol (final concentration of glycerol, 25%), aliquoted, and stored at –80°C until use (working stocks). Aliquots of the working stocks were inoculated in SP4 broth and cultured at 37°C for 4 to 12 h to reach the exponential phase based on the growth rates of the individual working stocks. After incubation, the culture medium was collected and centrifuged at 3,000 × *g* for 30 min at room temperature. The resulting *Ureaplasma* pellet was resuspended with 1 ml of SP4 broth and kept on ice until intra-amniotic inoculation (inoculation suspension). The *Ureaplasma* cell count in the inoculation suspension was measured using flow cytometry by following a modified version of a previously described method for counting *Mycoplasma* (65) with a bacterial counting kit (catalog no. L34856; Live/Dead *Bac*Light bacterial viability and counting kit for flow cytometry; Invitrogen by Thermo Fisher Scientific, Carlsbad, CA). Briefly, a fraction of the inoculation suspension (50 μl) was taken, diluted 10× with 1× PBS, and then stained with SYTO9 (final concentration, 10 μM) for 15 min at room temperature in the dark. Stained bacteria were centrifuged at 3,000 × *g* for 30 min at room temperature, resuspended with 500 μl of 1× PBS, and acquired using the BD LSR II flow cytometer (BD Bioscience, San Jose, CA) and BD FACSDiva 6.0 software (BD Bioscience). The absolute number of cells was determined using counting beads included in the kit. The *Ureaplasma* suspension was diluted with SP4 broth based on the obtained count. As the final yield of *Ureaplasma* cells varied in each inoculation suspension, those with final concentrations of *Ureaplasma* from ≥1 × 10^3^ to ≤1 × 10^4^ cells/25 μl were used for intra-amniotic inoculation of pregnant mice within a single study group (referred to as “low dose”), and those from >1 × 10^4^ to ≤1 × 10^5^ cells/25 μl were used for intra-amniotic inoculation of pregnant mice assigned to a second group (referred to as “high dose”). These doses were chosen based on concentrations of *Ureaplasma* species recovered from amniotic fluid of women with intra-amniotic infection. PBS and SP4 broth were used as the sham control and vehicle control, respectively. Since the intra-amniotic injection of SP4 broth alone caused increased concentrations of specific cytokines/chemokines in amniotic fluid (see Fig. S2) and upregulation of inflammatory genes in primary human amniocytes (Fig. S8), all experiments with *Ureaplasma* were compared to SP4 broth as the vehicle control treatment.

### Animal model of *Ureaplasma*-induced intra-amniotic infection.

Dams were anesthetized at 16.5 dpc by inhalation of 2% isoflurane (Aerrane; Baxter Healthcare Corporation, Deerfield, IL) and 1 to 2 liters/min of oxygen in an induction chamber. This gestational age (16.5 dpc) was chosen for its equivalency to the third trimester of human pregnancy, when preterm birth most frequently occurs (6, 66). Anesthesia was maintained with a mixture of 1.5 to 2% isoflurane and 1.5 to 2 liters/min of oxygen. Mice were positioned on a heating pad and stabilized with adhesive tape. Fur removal from the abdomen and thorax was achieved by applying Nair cream (Church & Dwight Co., Inc., Ewing, NJ) to those areas. Body temperature was maintained in the range of 37 ± 1°C and detected with a rectal probe (VisualSonics, Toronto, Ontario, Canada), and respiratory and heart rates were monitored by electrodes embedded in the heating pad. An ultrasound probe was fixed and mobilized with a mechanical holder, and the transducer was slowly moved toward the abdomen. Ultrasound-guided intra-amniotic inoculation with *Ureaplasma* (low dose or high dose) was performed in each amniotic sac using a 30G needle (BD PrecisionGlide needle; Becton Dickinson, Franklin Lakes, NJ). Controls were injected with 25 μl of SP4 broth or sterile 1× PBS. The syringe was stabilized by a mechanical holder (VisualSonics). After the ultrasound examination, the mice were placed under a heat lamp for recovery (defined as when the mouse resumed normal activities such as walking and responding), which typically occurred 10 to 20 min after removal from anesthesia. After recovery, mice were video-monitored to observe pregnancy outcomes.

### Video monitoring of pregnancy outcomes.

Pregnancy parameters, including the rates of preterm birth and pup mortality, were recorded via video camera (Sony Corporation, Tokyo, Japan). Preterm birth was defined as delivery occurring before 18.5 dpc, and its rate was represented by the percentage of females delivering preterm among the total number of mice injected. The rate of pup mortality for each litter was defined as the proportion of delivered pups found dead among the total litter size.

### Tissue sampling from dams intra-amniotically inoculated with *Ureaplasma* isolates.

Dams were intra-amniotically inoculated with 1 × 10^4^ cells/sac of *Ureaplasma* isolate 3 or *Ureaplasma* isolate 4, or injected with 25 μl of sterile 1× PBS or SP4 broth at 16.5 dpc, as described above. Mice were euthanized at 17.5 dpc (16 h post-inoculation), and maternal blood was collected by cardiac puncture and placed into a 1.5-ml Safe-Lock Eppendorf tube (Fisher Scientific, Hanover Park, IL). Serum (*n* = 6 each) was separated from the maternal peripheral blood and stored at −20°C until analysis. Animal dissection to obtain the maternal brain, maternal lung, maternal liver, maternal spleen, maternal para-aortic lymph node (PALN), decidua, uterus, cervix, fetal membranes, placenta, fetal brain, fetal lung, fetal thymus, fetal spleen, fetal liver, and fetal intestine (*n* = 6 each) was performed. Tissues were snap-frozen in liquid nitrogen and stored at –80°C until analysis or placed in RNA*later* stabilization solution (Invitrogen/Thermo Fisher Scientific) according to the manufacturer’s instructions. Amniotic fluid was also collected from each amniotic sac with a 26G needle and placed into a 0.5-ml Safe-Lock Eppendorf tube (Fisher Scientific). Amniotic fluid samples were centrifuged at 1,300 × *g* for 10 min at 4°C, and the supernatants were separated and stored at −20°C until analysis.

### Determination of cytokine concentrations in amniotic fluid and maternal serum.

Amniotic fluid and maternal serum samples were assessed for cytokine/chemokine concentrations. A ProcartaPlex mouse cytokine and chemokine panel 1A 36-plex (Invitrogen/Thermo Fisher Scientific) was used to measure the concentrations of IFNα, IFNγ, IL-12p70, IL-1β, IL-2, TNFα, granulocyte-macrophage colony-stimulating factor (GM-CSF), IL-18, IL-17A, IL-22, IL-23, IL-27, IL-9, IL-15/IL-15R, IL-13, IL-4, IL-5, IL-6, IL-10, Eotaxin (CCL11), IL-28, IL-3, LIF, IL-1α, IL-31, GRO-α (CXCL1), MIP-1α (CCL3), IP-10 (CXCL10), MCP-1 (CCL2), MCP-3 (CCL7), MIP-1β (CCL4), MIP-2 (CXCL2), RANTES (CCL5), G-CSF, M-CSF, and ENA-78 (CXCL5) in the serum and amniotic fluid samples, according to the manufacturer’s instructions. Plates were read by using the Luminex FLEXMAP 3D (Luminex, Austin, TX), and analyte concentrations were calculated with MILLIPLEX Analyst v5.1 Flex software (Vigene Tech, Carlisle, MA). The sensitivities of the assays were as follows: 3.03 pg/ml (IFNα), 0.09 pg/ml (IFNγ), 0.21 pg/ml (IL-12p70), 0.14 pg/ml (IL-1β), 0.10 pg/ml (IL-2), 0.39 pg/ml (TNFα), 0.19 pg/ml (GM-CSF), 9.95 pg/ml (IL-18), 0.08 pg/ml (IL-17A), 0.24 pg/ml (IL-22), 2.21 pg/ml (IL-23), 0.34 pg/ml (IL-27), 0.28 pg/ml (IL-9), 0.42 pg/ml (IL-15/IL-15R), 0.16 pg/ml (IL-13), 0.03 pg/ml (IL-4), 0.32 pg/ml (IL-5), 0.21 pg/ml (IL-6), 0.69 pg/ml (IL-10), 0.01 pg/ml (Eotaxin/CCL11), 20.31 pg/ml (IL-28), 0.11 pg/ml (IL-3), 0.28 pg/ml (LIF), 0.32 pg/ml (IL-1α), 0.45 pg/ml (IL-31), 0.05 pg/ml (GRO-α/CXCL1), 0.13 pg/ml (MIP-1α/CCL3), 0.26 pg/ml (IP-10/CXCL10), 3.43 pg/ml (MCP-1/CCL2), 0.15 pg/ml (MCP-3/CCL7), 1.16 pg/ml (MIP-1β/CCL4), 0.37 pg/ml (MIP-2/CXCL2), 0.35 pg/ml (RANTES/CCL5), 0.19 pg/ml (G-CSF), 0.02 pg/ml (M-CSF), and 5.67 pg/ml (ENA-78/CXCL5). Interassay and intra-assay coefficients of variation were less than 10%.

### Bacterial burden determination of *U. parvum* in maternal and fetal tissues and at the maternal-fetal interface by real-time quantitative PCR.

Total DNA was isolated from the frozen tissues of the maternal brain, maternal lung, maternal liver, maternal spleen, maternal PALN, decidua, uterus, cervix, fetal membranes, placenta, fetal brain, fetal lung, fetal thymus, fetal spleen, fetal liver, and fetal intestine by using DNeasy blood and tissue kits (Qiagen), according to the manufacturer’s instructions. DNA concentrations were assessed with a Qubit dsDNA HS assay kit and a Qubit 3 fluorometer (Invitrogen/Thermo Fisher Scientific) according to the manufacturer’s instructions.

U. parvum DNA abundances in the mouse tissues were measured by qPCR using taxon-specific primers (5′-CCAGGTAAATTAGTACCAGG-3′ and 5′-CCTGATGGAATATCGAAACG-3′) targeting the *ureB* gene, as previously described (67). Each 20-μl reaction contained 0.6 μM concentrations of each primer, 10.0 μl of 2× QuantiTect SYBR green PCR kit (Qiagen), and 3.0 μl of purified DNA diluted to a maximum concentration of 50.0 ng/μl. Cycling conditions for the assay were as follows: 95°C for 15 min, followed by 40 cycles of 94°C for 15 s, 55°C for 30 s, and 72°C for 25 s. All qPCRs and data collection were carried out in 96-well plates with an ABI 7500 Fast real-time PCR system using software v2.3 (Applied Biosystems, Alameda, CA). Duplicate reactions were carried out for all samples, and all samples were run across a total of 12 batches.

DNA derived from the clinical *U. parvum* isolate (isolate 1) was quantified using a Qubit 3.0 fluorometer with a Qubit dsDNA HS assay kit (Invitrogen/Thermo Fisher Scientific) and subsequently used for the generation of standard curves. It was estimated that the *U. parvum* isolate genome has a mass of 4.78 × 10^5^ kDa and contains a single copy of the *ureB* gene. A standard curve based on triplicate 10-fold serial dilutions ranging from 1.70 × 10^6^ to 1.70 × 10^1^ *ureB* gene copies was included in each qPCR run. The standard curves were used to evaluate the performance of the qPCR assay by estimation of its efficiency based on the slope of regression lines, according to a previous study (68). Raw amplification data were normalized to the ROX passive reference dye and analyzed using an automatic baseline setting and a threshold setting of 0.05. Quantification cycle (*C_q_*) values for samples were calculated based on the mean number of cycles required for normalized fluorescence to exponentially increase.

After plotting a regression of log(*ureB* gene copy number) and *C_q_* values for standard curves included in each qPCR run, the *ureB* gene copy number in mouse samples was calculated using the equation *X_o_* = *E_AMP_*^(^*^b^*^–^*^Cq^*^)^, where *E_AMP_* is the exponential amplification value for the qPCR assay, calculated as *E_AMP_* = 10^(−1/^*^m^*^)^, and *b* and *m* are the intercept and slope of the regression (69).

### RNA isolation, cDNA synthesis, and reverse transcription-quantitative PCR analysis of murine tissues.

Total RNA was isolated from the decidua, uterus, cervix, fetal membranes, placenta, fetal brain, fetal lung, fetal thymus, fetal spleen, fetal liver, and fetal intestine using QIAshredders, RNase-free DNase sets, and RNeasy Mini kits (all from Qiagen) according to the manufacturer’s instructions. RNA concentrations and integrity were evaluated with the Bioanalyzer 2100 (Agilent Technologies, Wilmington, DE). Complementary (c)DNA was synthesized by using SuperScript III first-strand synthesis supermix (Invitrogen/Thermo Fisher Scientific). Gene expression profiling was performed on the BioMark system for high-throughput RT-qPCR (Fluidigm, San Francisco, CA) with the TaqMan gene expression assays (Applied Biosystems/Life Technologies Corporation, Foster City, CA) listed in Table S2.

10.1128/mBio.00797-20.10 TABLE S2 Mouse and human primers. Download Table S2, DOCX file, 0.02 MB. Copyright © 2020 Motomura et al. 2020 Motomura et al. This content is distributed under the terms of the Creative Commons Attribution 4.0 International license.

### Primary cell culture of human amnion epithelial cells.

Immediately after collection, the amnion membrane was manually peeled from the underlying chorion layer of the chorioamniotic membranes and then dissected into small pieces. The amnion fragments were rinsed in 0.05% (wt/vol) trypsin/EDTA (Life Technologies) and incubated with 15 ml of fresh trypsin-EDTA at 37°C with gentle shaking for 10 min. The trypsin digestion supernatant was discarded, and the amnion fragments were placed into fresh trypsin-EDTA solution and amnion epithelial cells (AECs) were obtained by digesting at 37°C for 40 min with gentle shaking. The total digestion/incubation process was repeated twice. Fetal bovine serum (FBS; Thermo Fisher Scientific) was added to the supernatant to stop digestion between each incubation period. Finally, digested tissues were filtered through a 100-μm cell strainer (Fisher Scientific, Durham, NC). The resulting cell suspensions were centrifuged at 300 × *g* for 10 min, and the cells were cultured in Dulbecco modified Eagle medium (Life Technologies) containing 10% FBS and 100 U/ml penicillin and streptomycin (Thermo Fisher Scientific) at 37°C with 5% CO_2_. The absence of mycoplasma contamination in AEC cultures was confirmed by qPCR using the MycoSensor QPCR assay kit (catalog no. 302107; Agilent Technologies, Santa Clara, CA). Immunofluorescence staining was performed with cells at passage 1. Cells at passage 2 were used for incubation with *Ureaplasma* isolates.

### Immunofluorescence and confocal microscopy.

Amnion epithelial cells were cultured in four-well Lab-Tek chamber slides (Nunc, Rochester, NY) at 1 × 10^4^ cells per well. Next, cells were fixed using 4% paraformaldehyde (Electron Microscopy Sciences Hatfield, PA) for 20 min at room temperature, rinsed with 1× PBS, and permeabilized using 0.25% Triton X-100 (EMD Millipore, Billerica, MA) for 5 min at room temperature. After 30 min of incubation with an Image-iT Fx signal enhancer (Invitrogen by Thermo Fisher Scientific), nonspecific antibody interactions were blocked using antibody diluent/block (catalog no. ARD1001EA; Perkin-Elmer, Boston, MA) for 30 min at room temperature. The cells were then stained with a mouse anti-human EpCAM (catalog no. MA5-12436; Invitrogen) antibody at room temperature for 1 h. Mouse IgG (Invitrogen/Thermo Fisher Scientific) was used as a negative control. After staining, the cells were washed with 1× PBS containing 0.1% Tween 20 (PBST; Sigma-Aldrich, Saint Louis, MO). After a wash step, secondary goat anti-mouse IgG–Alexa Fluor 594 (catalog no. A11032; Life Technologies) and Alexa Fluor 488 phalloidin (catalog no. A12379; Life Technologies) were added, and the cells were incubated for 1 h at room temperature in the dark. Finally, the cells were washed with PBST and mounted using ProLong Diamond antifade mountant with DAPI (4′,6′-diamidino-2-phenylindole; Life Technologies). Immunofluorescence was visualized using a Zeiss LSM 780 laser scanning confocal microscope (Carl Zeiss Microscopy, Jena, Germany) at the Microscopy, Imaging, and Cytometry Resources Core at the Wayne State University School of Medicine (http://micr.med.wayne.edu/).

### Determination of inflammatory genes in primary amnion epithelial cells.

Amnion epithelial cells were plated into six-well tissue culture plates (Corning, Inc., Corning, NY) at 1.5 × 10^5^ cells/well and cultured at 37°C with 5% CO_2_. The cells were then incubated with *Ureaplasma* isolate 3 or isolate 4 (1.3 × 10^5^ cells/well with serum-free Opti-MEM [Life Technologies]), sterile 1× PBS with serum-free Opti-MEM, or SP4 broth with serum-free Opti-MEM as controls. After 24 h of treatment, total RNA was isolated from cells by using the RNeasy Mini kits (Qiagen) according to the manufacturer’s instructions. RNA concentrations were assessed with a NanoDrop 1000 spectrophotometer (Thermo Fisher Scientific). cDNA was synthesized using a high-capacity cDNA reverse transcription kit with RNase inhibitor (Thermo Fisher Scientific, Waltham, MA), according to the manufacturer’s instructions. cDNA was amplified using TaqMan PreAmp master mix on an ABI 7500 Fast real-time PCR system. For all genes besides NLRP7, mRNA expression was determined by qPCR utilizing a BioMark high-throughput qRT-PCR system (Fluidigm) with the TaqMan gene expression assays (Applied Biosystems) listed in Table S2. For NLRP7, mRNA expression was determined by qPCR using an ABI 7500 Fast real-time PCR system with the TaqMan gene expression assays.

### Clarithromycin treatment of mice intra-amniotically inoculated with *Ureaplasma parvum*.

Dams were intra-amniotically inoculated with 1 × 10^4^ cells/25 μl of *U. parvum* (isolate 4) in each amniotic sac under ultrasound guidance at 16.5 dpc and then monitored via video camera until delivery in order to obtain the gestational age and rate of preterm birth. The dams were injected subcutaneously with 75 mg/kg of clarithromycin (CLR; Sigma-Aldrich) or its vehicle control (dimethyl sulfoxide [DMSO]) at 6, 18, 30, 54, and 78 h after intra-amniotic inoculation with *U. parvum*. Clarithromycin (reconstituted with DMSO) or DMSO (vehicle control) was diluted with sterile 5% dextrose water. The dose of CLR was determined based on a previous study (70) showing that a 75 mg/kg/day dose of CLR in mice was equivalent to human oral CLR dosages (ranging from 250 mg twice daily to 500 mg twice daily in adults [71]). The latter dose has been demonstrated to eradicate intra-amniotic infection or inflammation in a subset of women with preterm labor and intact membranes (23), cervical insufficiency (24), or preterm prelabor rupture of the membranes (22) when it is administered together with other antibiotics (ceftriaxone and metronidazole).

### Statistical analysis.

Statistical analyses were performed using Prism v8 (GraphPad, San Diego, CA), the R package (https://www.r-project.org/), or Subio Platform v1.23.5551 (Subio, Inc., Kagoshima, Japan). To determine rates of preterm birth, a Fisher's exact test was used. Kaplan-Meier survival curves were used to plot and compare the gestational length data (a Gehan-Breslow-Wilcoxon test). The unpaired *t* test was performed for the statistical analysis of neonatal mortality. For multiplex assays, the concentrations of the cytokines/chemokines were log transformed and statistically tested by one-way analysis of variance (ANOVA), followed by a Tukey’s test to determine differences between groups. The statistical difference of the *ureB* gene copies between isolate 3 and isolate 4 was tested by the Mann-Whitney U test. For qPCR analysis in mouse tissues and AECs, negative delta threshold cycle (–Δ*C_T_*) values were determined using multiple reference genes (*Gusb*, *Hsp90ab1*, *Gapdh*, and *Actb* for mouse tissues or *RPLP0*, *GAPDH*, and *ACTB* for AECs) averaged within each sample to determine gene expression levels. In mouse tissues, the –Δ*C_T_* values were normalized by calculating the Z-score of each gene, and then heatmaps were created, which represent the mean of the Z-score of –Δ*C_T_* and the hierarchical clustering using uncentered correlation. Relative fold changes for mRNA expression were calculated using the 2^–ΔΔ^*^CT^* method (72) and as shown as the log_2_(fold change). One-way ANOVA, followed by a Tukey’s test, was performed to assess the statistical significance of group comparisons for mRNA expression. A *P* value of <0.05 was considered statistically signiﬁcant.

### Data availability.

Most relevant data are present within the manuscript and in the supplemental material.

Mouse raw data may be requested from the corresponding author. *Ureaplasma* genome sequence files have been deposited to the NCBI Sequence Read Archive under the BioProject accession number PRJNA615648 (https://www.ncbi.nlm.nih.gov/bioproject/PRJNA615648).
