# Supplementary material for: Intra-Amniotic Infection with Ureaplasma parvum Causes Preterm Birth and Neonatal Mortality That Are Prevented by Treatment with Clarithromycin
Source: mBio. 2020 Jun 23;11(3):e00797-20. doi: 10.1128/mBio.00797-20 (PMC7315120; doi:10.1128/mBio.00797-20)
Supplement: TABLE S1 [file mBio.00797-20-st001.docx]

**Table S1. Clinical and demographic characteristics of women from whose amniotic fluid *Ureaplasma* species were isolated.**

|  | **Isolate 1** | **Isolate 2** | **Isolate 3** | **Isolate 4** |
| --- | --- | --- | --- | --- |
| Maternal age (years) | 19 | 24 | 21 | 37 |
| Body mass index (kg/m^2^) | 20.1 | 31.4 | 30.7 | 35.9 |
| Gravida | 2 | 3 | 1 | 6 |
| Race/Ethnicity | African American | African American | African American | African American |
| Gestational age at amniocentesis (weeks, days) | 39w4d | 39w6d | 40w6d | 20w0d |
| IL-6 (ng/mL) | 3.5 | 73.6 | 51.1 | 29.9 |
| Amniotic Fluid Glucose (mg/dl) | 1 | N/A | 1 | 31 |
| Amniotic Fluid WBC (cells/mm^3^) | 590 | N/A | 2600 | 3 |
| Gestational age at delivery (weeks/days) | 39w4d | 40w0d | 40w6d | 30w6d |
| Mode of delivery | Vaginal | Cesarean section | Vaginal | Vaginal |
| Birthweight (grams) | 3,005 | 3,360 | 3,975 | 1,680 |
| Acute maternal inflammatory response* | Stage 1 | Stage 2 | Stage 1 | Stage 2 |
| Acute fetal inflammatory response** | Stage 1 | Stage 2 | Stage 1 | Stage 3 |

*Acute maternal inflammatory response

Stage 1 (Early acute subchorionitis or chorionitis)

Stage 2 (Acute chorioamnionitis)

Stage 3 (Necrotizing chorioamnionitis)

**Acute fetal inflammatory response

Stage 1 (Chorionic vasculitis or umbilical phlebitis)

Stage 2 (Umbilical arteritis)

Stage 3 (Necrotizing funisitis)

N/A: Not analyzed
