# Supplementary material for: Intra-Amniotic Infection with Ureaplasma parvum Causes Preterm Birth and Neonatal Mortality That Are Prevented by Treatment with Clarithromycin
Source: mBio. 2020 Jun 23;11(3):e00797-20. doi: 10.1128/mBio.00797-20 (PMC7315120; doi:10.1128/mBio.00797-20)
Supplement: TABLE S2 [file mBio.00797-20-st002.docx]

| **Table S2. Mouse and human primers** |  |  |
| --- | --- | --- |
| **Mouse primers** | | |
| **Name** | **Symbol** | **Assay ID** |
| **Reference genes** | | |
| Actin, beta | *Actb* | Mm04394036_g1 |
| Glucuronidase, beta | *Gusb* | Mm01197698_m1 |
| Glyceraldehyde-3-phosphate dehydrogenase | *Gapdh* | Mm99999915_g1 |
| Heat shock protein 90 alpha (cytosolic), class B member 1 | *Hsp90ab1* | Mm00833431_g1 |
| **Inflammatory genes** | | |
| Absent in melanoma 2 | *Aim2* | Mm01295719_m1 |
| Arachidonate 15-lipoxygenase | *Alox15* | Mm00507789_m1 |
| Arginase, liver | *Arg1* | Mm00475988_m1 |
| Caspase recruitment domain family member 10 | *Card10* | Mm00459941_m1 |
| Caspase recruitment domain family member 11 | *Card11* | Mm01201965_m1 |
| Caspase recruitment domain family member 14 | *Card14* | Mm00459947_m1 |
| Caspase recruitment domain family member 6 | *Card6* | Mm01297056_m1 |
| Caspase 1 | *Casp1* | Mm00438023_m1 |
| Caspase 8 | *Casp8* | Mm01255716_m1 |
| Chemokine (C-C motif) ligand 17 | *Ccl17* | Mm01244826_g1 |
| Chemokine (C-C motif) ligand 2 | *Ccl2* | Mm00441242_m1 |
| Chemokine (C-C motif) ligand 22 | *Ccl22* | Mm00436439_m1 |
| Chemokine (C-C motif) ligand 24 | *Ccl24* | Mm00444701_m1 |
| Chemokine (C-C motif) ligand 3 | *Ccl3* | Mm00441259_g1 |
| Chemokine (C-C motif) ligand 5 | *Ccl5* | Mm01302427_m1 |
| CD3 antigen, epsilon polypeptide | *Cd3e* | Mm01179194_m1 |
| Chitinase-like 3 | *Chil3* | Mm00657889_mH |
| Cytotoxic T-lymphocyte-associated protein 4 | *Ctla4* | Mm00486849_m1 |
| Chemokine (C-X-C motif) ligand 1 | *Cxcl1* | Mm04207460_m1 |
| Chemokine (C-X-C motif) ligand 10 | *Cxcl10* | Mm00445235_m1 |
| Chemokine (C-X-C motif) ligand 9 | *Cxcl9* | Mm00434946_m1 |
| Defensin alpha 1 | *Defa1* | Mm02524428_g1 |
| Defensin beta 1 | *Defb1* | Mm00432803_m1 |
| Gasdermin D | *Gsdmd* | Mm00509958_m1 |
| High mobility group box 1 | *Hmgb1* | Mm00849805_gH |
| Intercellular adhesion molecule 1 | *Icam1* | Mm00516023_m1 |
| Intercellular adhesion molecule 2 | *Icam2* | Mm00494862_m1 |
| Interferon gamma | *Ifng* | Mm01168134_m1 |
| Interleukin-10 | *Il10* | Mm01288386_m1 |
| Interleukin-10 receptor alpha | *Il10ra* | Mm00434151_m1 |
| Interleukin-10 receptor beta | *Il10rb* | Mm00434157_m1 |
| Interleukin-12a | *Il12a* | Mm00434169_m1 |
| Interleukin-12b | *Il12b* | Mm01288989_m1 |
| Interleukin-17A | *Il17a* | Mm00439618_m1 |
| Interleukin-18 | *Il18* | Mm00434226_m1 |
| Interleukin-1 alpha | *Il1a* | Mm00439620_m1 |
| Interleukin-1 beta | *Il1b* | Mm00434228_m1 |
| Interleukin-1 receptor type 1 | *Il1r1* | Mm00434237_m1 |
| Interleukin-2 | *Il2* | Mm00434256_m1 |
| Interleukin-23a | *Il23a* | Mm00518984_m1 |
| Interleukin-27 | *Il27* | Mm00461162_m1 |
| Interleukin-3 | *Il3* | Mm00439631_m1 |
| Interleukin-33 | *Il33* | Mm00505403_m1 |
| Interleukin-4 | *Il4* | Mm00445259_m1 |
| Interleukin-5 | *Il5* | Mm00439646_m1 |
| Interleukin-6 | *Il6* | Mm00446190_m1 |
| Interleukin-9 | *Il9* | Mm00434305_m1 |
| Interferon regulatory factor 4 | *Irf4* | Mm00516431_m1 |
| Interferon regulatory factor 5 | *Irf5* | Mm00496477_m1 |
| Mannose-binding lectin (protein C) 2 | *Mbl2* | Mm00487623_m1 |
| Antigen identified by monoclonal antibody Ki 67 | *Mki67* | Mm01278617_m1 |
| Myeloid differentiation primary response gene 88 | *Myd88* | Mm00440338_m1 |
| Nuclear factor of kappa light polypeptide gene enhancer in B cells 1 | *Nfkb1* | Mm00476361_m1 |
| Nuclear factor of kappa light polypeptide gene enhancer in B cells 2 | *Nfkb2* | Mm00479807_m1 |
| Nuclear factor of kappa light polypeptide gene enhancer in B cells inhibitor, zeta | *Nfkbiz* | Mm00600522_m1 |
| NLR family, CARD domain containing 4 | *Nlrc4* | Mm01233151_m1 |
| NLR family, CARD domain containing 5 | *Nlrc5* | Mm01243039_m1 |
| NLR family, pyrin domain containing 10 | *Nlrp10* | Mm00615833_m1 |
| NLR family, pyrin domain containing 12 | *Nlrp12* | Mm01329688_m1 |
| NLR family, pyrin domain containing 1A | *Nlrp1a* | Mm03047263_m1 |
| NLR family, pyrin domain containing 2 | *Nlrp2* | Mm00624616_m1 |
| NLR family, pyrin domain containing 3 | *Nlrp3* | Mm00840904_m1 |
| NLR family, pyrin domain containing 6 | *Nlrp6* | Mm00460229_m1 |
| NLR family, pyrin domain containing 9B | *Nlrp9b* | Mm01312681_g1 |
| Nucleotide-binding oligomerization domain containing 1 | *Nod1* | Mm00805062_m1 |
| Nucleotide-binding oligomerization domain containing 2 | *Nod2* | Mm00467543_m1 |
| Nitric oxide synthase 2, inducible | *Nos2* | Mm00440502_m1 |
| Purinergic receptor P2X, ligand-gated ion channel, 7 | *P2rx7* | Mm01199500_m1 |
| PYD and CARD domain containing | *Pdcd1* | Mm01285676_m1 |
| PYD and CARD domain containing | *Pycard* | Mm00445747_g1 |
| Resistin like alpha | *Retnla* | Mm00445109_m1 |
| SR-related CTD-associated factor 11 | *Scaf11* | Mm01297328_m1 |
| Selectin, endothelial cell | *Sele* | Mm00441278_m1 |
| Selectin, lymphocyte | *Sell* | Mm00441291_m1 |
| Suppressor of cytokine signaling 1 | *Socs1* | Mm00782550_s1 |
| Suppressor of cytokine signaling 2 | *Socs2* | Mm01236704 m1 |
| Signal transducer and activator of transcription 1 | *Stat1* | Mm01257286_m1 |
| Signal transducer and activator of transcription 6 | *Stat6* | Mm01160477_m1 |
| Transforming growth factor, beta 1 | *Tgfb1* | Mm01178820_m1 |
| Toll-like receptor 2 | *Tlr2* | Mm00442346_m1 |
| Toll-like receptor 4 | *Tlr4* | Mm00445273_m1 |
| Toll-like receptor 9 | *Tlr9* | Mm00446193_m1 |
| Tumor necrosis factor | *Tnf* | Mm00443258_m1 |
| Tumor necrosis factor receptor superfamily member 1a | *Tnfr1* | Mm00441883_g1 |
| Tumor necrosis factor receptor superfamily member 1b | *Tnfr2* | Mm00441889_m1 |
| Vascular cell adhesion molecule 1 | *Vcam1* | Mm01320970_m1 |
| **Contractility-related genes** | | |
| Oxytocin receptor | *Oxtr* | Mm01182684_m1 |
| Prostaglandin F receptor | *Ptgfr* | Mm00436055_m1 |
| Prostaglandin-endoperoxide synthase 2 | *Ptgs2* | Mm00478374_m1 |
| Gap junction protein, alpha 1 | *Gja1* | Mm00439105_m1 |
|  |  |  |
| **Human primers** | | |
| **Reference genes** | | |
| Actin, beta | *ACTB* | Hs99999903_m1 |
| Glyceraldehyde-3-phosphate dehydrogenase | *GAPDH* | Hs99999905_m1 |
| Ribosomal protein lateral stalk subunit P0 | *RPLP0* | Hs99999902_m1 |
| **Inflammatory genes** | | |
| Absent in melanoma 2 | *AIM2* | Hs00915710_m1 |
| Caspase-1 / Interleukin-1 converting enzyme | *CASP1* | Hs00354836_m1 |
| Caspase-4 | *CASP4* | Hs01031947_m1 |
| Defensin alpha 1 | *DEFA1* | Hs00234383_m1 |
| Defensin beta 1 | *DEFB1* | Hs00608345_m1 |
| Gasdermin D | *GSDMD* | Hs00986739_g1 |
| Interleukin-18 | *IL18* | Hs01038788_m1 |
| Interleukin-1 alpha | *IL1A* | Hs00174092_m1 |
| Interleukin-1 beta | *IL1B* | Hs00174097_m1 |
| Interleukin-6 | *IL6* | Hs00174131_m1 |
| Nuclear factor of kappa light polypeptide gene enhancer in B cells 1 | *NFKB1* | Hs00765730_m1 |
| NLR family CARD domain-containing protein 4 | *NLRC4* | Hs00368367_m1 |
| NACHT, LRR and PYD domains-containing protein 1 | *NLRP1* | Hs00248187_m1 |
| NACHT, LRR and PYD domains-containing protein 3 | *NLRP3* | Hs00918082_m1 |
| NACHT, LRR and PYD domains-containing protein 7 | *NLRP7* | Hs00373683_m1 |
| Nucleotide-binding oligomerization domain-containing protein 1 | *NOD1* | Hs00196075_m1 |
| Nucleotide-binding oligomerization domain-containing protein 2 | *NOD2* | Hs00223394_m1 |
| PYD and CARD domain containing | *PYCARD* | Hs01547324_gH |
| Toll-like receptor 2 | *TLR2* | Hs00610101_m1 |
| Toll-like receptor 4 | *TLR4* | Hs00152939_m1 |
| Toll-like receptor 9 | *TLR9* | Hs00370913_s1 |
| Tumor necrosis factor | *TNF* | Hs00174128_m1 |
